# Ensemble Learning-Based Approach for Gas Detection Using an Electronic Nose in Robotic Applications

**DOI:** 10.3389/fchem.2022.863838

**Published:** 2022-04-28

**Authors:** Han Fan, Erik Schaffernicht, Achim J. Lilienthal

**Affiliations:** Mobile Robotics & Olfaction Lab, Centre for Applied Autonomous Sensor Systems (AASS), Örebro University, Örebro, Sweden

**Keywords:** electronic nose, metal oxide semiconductor sensor, gas detection, gas sensing, open sampling systems, ensemble learning, robotic olfaction

## Abstract

Detecting chemical compounds using electronic noses is important in many gas sensing related applications. A gas detection system is supposed to indicate a significant event, such as the presence of new chemical compounds or a noteworthy change of concentration levels. Existing gas detection methods typically rely on prior knowledge of target analytes to prepare a dedicated, supervised learning model. However, in some scenarios, such as emergency response, not all the analytes of concern are a priori known and their presence are unlikely to be controlled. In this paper, we take a step towards addressing this issue by proposing an ensemble learning based approach (ELBA) that integrates several one-class classifiers and learns online. The proposed approach is initialized by training several one-class models using clean air only. During the sampling process, the initialized system detects the presence of chemicals, allowing to learn another one-class model and update existing models with self-labelled data. We validated the proposed approach with real-world experiments, in which a mobile robot equipped with an e-nose was remotely controlled to interact with different chemical analytes in an uncontrolled environment. We demonstrated that the ELBA algorithm not only can detect gas exposures but also recognize baseline responses under a suspect short-term sensor drift condition. Depending on the problem setups in practical applications, the present work can be easily hybridized to integrate other supervised learning models when the prior knowledge of target analytes is partially available.

## 1 Introduction

Portable, low-cost electronic noses (e-noses) based on an array of partially selective MOX sensors are widely used for gas sensing in many applications, including but not limited to landfill (Perera et al., 2001; De Vito et al., 2011; Gębicki et al., 2014) and ship emission monitoring (Yuan et al., 2020), chemical leakage detection in industrial sites (Capelli et al., 2014; Bourne et al., 2020), early fire detection (Young et al., 2003; Scorsone et al., 2006; Joseph et al., 2015), exploration of interested areas for emergency response or environmental monitoring (He et al., 2019; Anyfantis et al., 2021), etc (Monroy and Gonzalez-Jimenez, 2019; Burgués and Marco, 2020a). In many real-world gas sensing related applications, gas detection comes as a fundamental task that recognizes the presence of gases by monitoring their concentration levels exceeding pre-defined thresholds. Furthermore, gas detection can also indicate significant events, such as the presence of new chemical compounds or a noteworthy change of concentration levels.

Due to the need for rapid, continuous sampling at varying locations in some application scenarios, gas detection is performed with open sampling systems (OSS), in which gas sensors are directly exposed to the uncontrolled environment. For example, ground robots or drones equipped with e-noses are increasingly used to carry out environmental monitoring tasks with no control over the sensing conditions (Bennetts et al., 2016; Fan et al., 2019b; Palacín et al., 2019). Some of the real-world applications, e.g., emergency responses to hazardous chemicals, are particularly complex and demanding for open sampling systems as they might deal with unknown analytes. In addition, although gas detection can be a standalone use to provide information about the presence of different analytes, it might be required to support further analysis on the identities, quantities and locations of detected chemicals. Consequently, gas detection should be integrated with gas discrimination or/and gas distribution mapping in an integrated pipeline instead of being a standalone task. In such cases, gas detection typically is followed by a subsequent gas discrimination task, which not only determines the presence of chemicals but also extracts recognizable measurements. Put another way, the gas detection task concerned here is performed without a known limit of detection (LOD) (Currie, 1995). Below we explain that this problem set-up leads to a few challenges.

**FIGURE 1:**
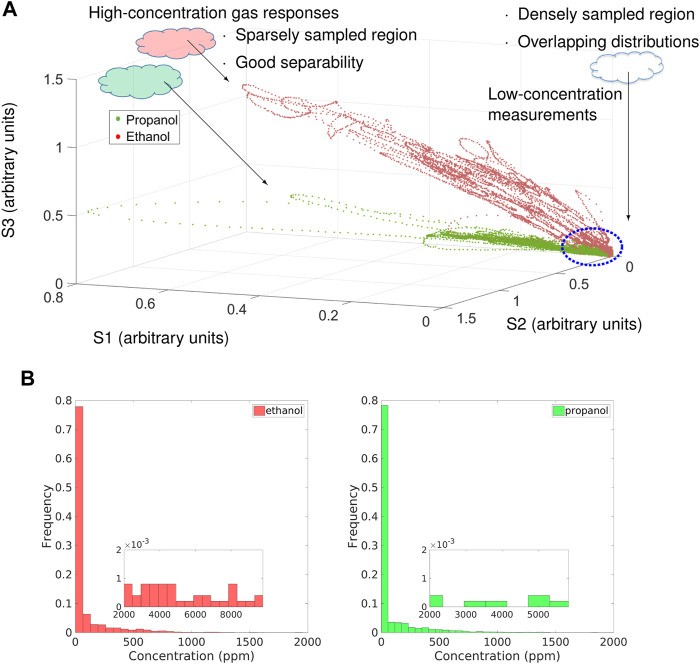
Feature space plot and the gas concentration histograms of measurements of propanol (green) and ethanol (red) collected in an uncontrolled environment with three E2V manufactured metal oxide (MOX) gas sensors (MICS-2710, MICS-5121, MICS-5521). **(A)** Feature space plot. Each data point is an instantaneous sensor response. **(B)** Concentration levels shown in the histograms were obtained by a photoionization detector (PID). The nested figures are the zoomed-in histograms at high concentrations. Both data of propanol and ethanol are clearly unbalanced with respect to the gas concentration.

It is often the case that the data sets of gas sensor responses in uncontrolled environments are unbalanced in terms of concentration levels. Figure 1A is a visualization of data points acquired by an open sampling system in 3D feature space. The concentration distributions of both classes are shown in Figure 1B. In the feature space plot, relatively high-concentration measurements have good separability for further gas identification or discrimination, but they are sparsely sampled, whereas low-concentration measurements are densely sampled. Such observation is also reflected in the concentration distributions shown in Figure 1B. Measurements of lower concentration levels are diluted with a large amount of clean air, making their responses pattern close to that of baseline responses. In the feature space, diluted measurements overlap for different compounds, showing poor separability, and therefore are less recognizable for gas discrimination than high-concentration measurements.

In other words, real-world working environments pose a series of challenges to gas detection using an e-nose. For the detection and extraction of recognizable measurements, the issue becomes critical in the two following aspects:

• a gas exposure reflected by gas sensor responses is not trivial to be captured as clearly segmented rising, steady-state and recovery phases, making the conventional three-phase sampling strategy (Trincavelli et al., 2009) inapplicable;
• gas detection solutions based on setting up thresholds for sensor responses become inaccurate.

The former is due to uncontrolled environmental conditions, and the latter is concerning as the possible presence of compounds is not a priori known.

**FIGURE 2:**
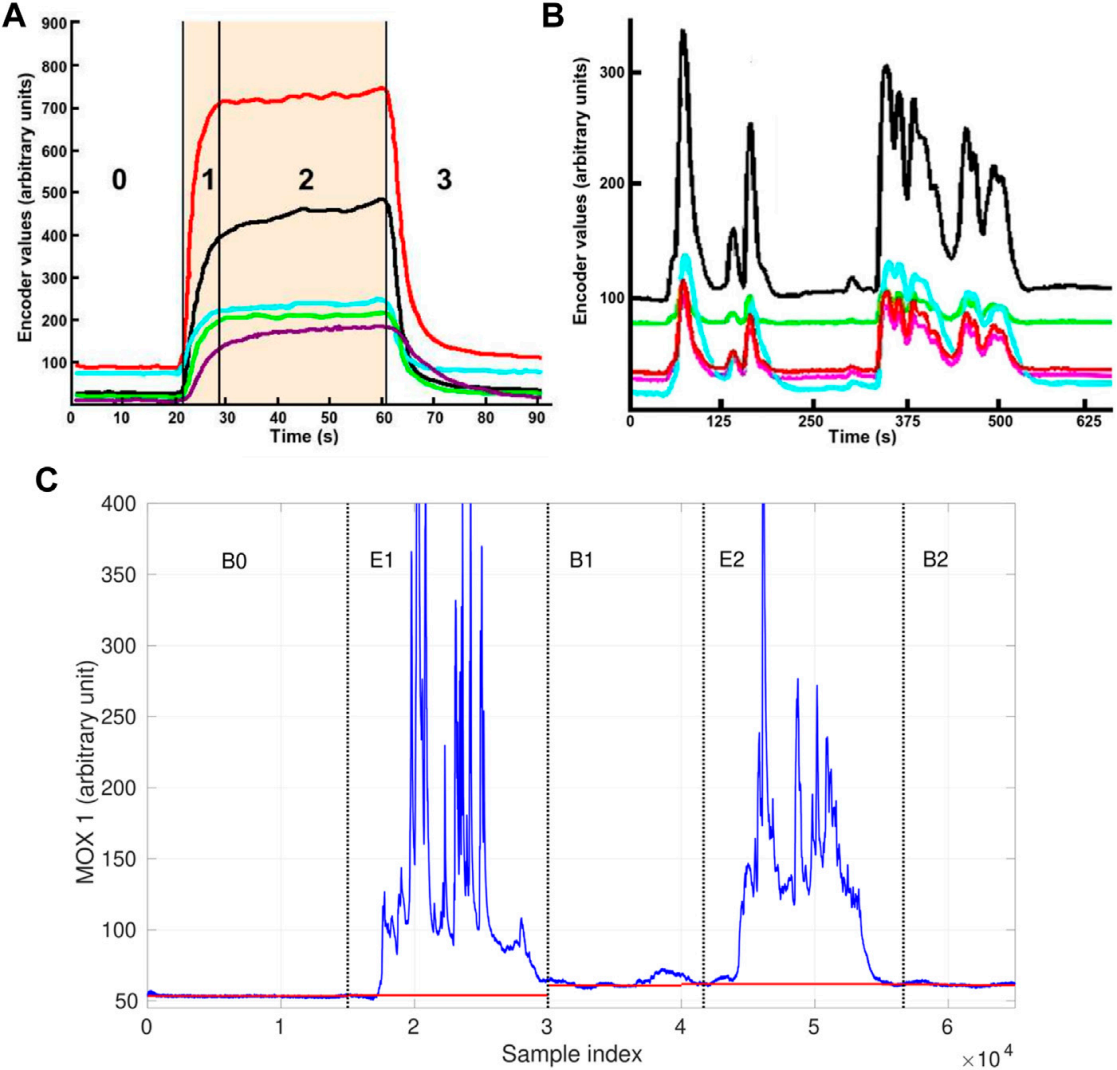
Responses patterns acquired with MOX sensor arrays with different sampling processes. **(A)** An example of the three-phase sampling process. The numbers in the figure indicate the stages of the sensor responses, namely 0-baseline response, 1-rising edge, 2-steady state, 3-recovery edge. The shaded area denotes the period of time during which the sensors are exposed to the chemical analyte, and each color represents the response of a single sensor. **(B)** Response pattern acquired with an open sampling system. Both images are adapted from (Trincavelli et al., 2009) with the author’s permission. **(C)** An example of baseline drift of a MOX sensor response of an open sampling system. The red segments represent the observed baseline offsets that correspond to the sensor response level under clean air. The baseline offsets in the periods B1 and B2 are higher than the initial baseline offset in the period B0.

Regarding the first aspect, uncontrolled conditions in open environments introduce gas concentration changes with fast dynamics, which prevent the use of the three-phase sampling strategy (Trincavelli et al., 2009) that is well established in laboratory-based applications, e.g., in food & beverage industry. In a three-phase sampling process, the sensors are first exposed to a reference gas (e.g., clean air) to establish a known baseline response level for the sensor array. Then, the sensors interact with injected gas samples under largely constant conditions over a prolonged time until a steady response state is reached. The sampling process concludes as the sensors recover to their baseline levels when the gas sample is flushed away. Figure 2A shows an example of sensor responses in a clear three-phase profile. In this case, the gas sensors inside a chamber have been exposed to the gas sample for a considerable amount of time. The closed chamber ensures that humidity, temperature, airflow, and gas exposure patterns are tightly controlled. Contrary to laboratory conditions, in uncontrolled environments, e-noses are typically directly exposed to dynamically changing conditions. These complex ambient conditions, as well as the turbulence and advection in gas dispersal or the movement of the sensing platform, cause fluctuating gas concentration levels, and the sensor responses show intermittent and transient behaviour instead of well-defined three-phase patterns. Figure 2B is an example in which the measurements are taken by sensors mounted on a mobile robot exploring in a large room with an e-nose. An obvious difference between Figures 2A,B is that, the sensor responses in Figure 2B never reached a steady state.

Second, it is challenging to perform gas detection on diluted measurements by thresholding. This is because if the gas identity is unknown, it is difficult to accurately determine its concentration levels from MOX sensor responses in absolute gas concentration units, e.g., ppm. When sensors are calibrated with several specific gases, the typical procedure is to define corresponding thresholds based on the known dose-response relationship to discriminate recognizable gas responses and baseline responses. However, these thresholds are not referential for unidentified measurements. Besides, the baseline responses of MOX sensors might drift over time during the sampling process. When sensor drift occurs, diluted measurements present a different pattern that is not identical with previously observed clean air, which creates an issue for the essential signal processing procedure on the instantaneous sensor responses, i.e. baseline correction. As suggested in (Hines et al., 1999), differential baseline correction can compensate for noise and inherently large or small signals of MOX gas sensor responses. In this correction method, the mean value of the baseline responses is extracted as the baseline offset. However, in uncontrolled environments, this offset may not remain constant due to the effects of temperature, humidity (Rudnitskaya, 2018), and short-term sensor drift. Such short-term drift is produced by the alteration of the MOX sensitive layer caused by molecule adsorption or fluctuations of the temperature and humidity during the gas exposition phase. The most effective solution to compensate for short-term drift is to have a time-consuming (tens of minutes) cleaning process after each gas exposure (Ahmadou et al., 2017), which is equivalent to repeated re-calibration. Since a continuous and rapid sampling process is often desired for open sampling systems, an alternative countermeasure is necessary. An example of short-term drift is shown in Figure 2C. In this exemplary data set, a MOX sensor was exposed to two chemical compounds separately in the period E1, E2 and was exposed to clean air in the period B0, B1, B2. As is shown, the initial baseline observed in the beginning (B0) was not recovered in B1 or B2, so the actual baseline offset drifted. In consequence, gas detection relying on the initial baseline offset is prone to declare diluted measurements as gas exposure. For applications where open sampling systems are deployed, the short-term drift is arguably a more critical issue than the long-term sensor drift due to contamination or ageing of the sensors. Although both drift behaviours affect the response patterns and increase the difficulty of accurate concentration quantification and gas discrimination, long-term drift is easier to be minimized by off-field procedures, such as periodic calibration and sensor replacement.

Previous works on gas detection using an e-nose typically take the strategy of modelling the MOX sensor responses to target gases. The modelling focus can be on the dynamic analysis for change point detection (Bordignon and Scagliarini, 2000; Pashami et al., 2012; Alavi-Shoshtari et al., 2018) or on the regression between the gas concentrations and sensor responses (Khalaf et al., 2009; Lentka et al., 2015). The applicability and quality of these supervised models rely on the availability of representative training data, which themselves must be free from sensor drift. (Perera et al., 2006), (Liu et al., 2018) and (Martinelli et al., 2013) addressed gas detection under sensor drift. An important common feature of these works is that their models are updated with new, self-labelled measurements so that the drifted sensor responses can be included. The idea of having an adaptive learning model by retraining with new data is also taken in the proposed approach.

To sum up, a critical challenge for gas detection in unknown and uncontrolled environments is to overcome the lack of a predefined, fixed determination threshold, and to identify which measurements are recognizable for the subsequent discriminative process in the meantime. In this paper, we propose an Ensemble Learning-Based Approach (ELBA) for gas detection to address this issue. This approach does not assume that the response patterns of the target analytes are known. Instead, it is initialized with a set of clean air measurements and then creates an ensemble of models learning from clean air and gas exposure based on different principles. The method has been validated with real-world MRO data sets that are affected by short-term sensor drift.

## 2 Methods

### 2.1 Overview of the Model Learning Process

The proposed ELBA is an ensemble learning system that comprises several one-class models to detect gas exposure events and learn the baseline response pattern. Using an ensemble of diverse models aims to improve the detection of recognizable measurements and, therefore, to benefit a subsequent gas discrimination stage (Kuncheva, 2014).

**FIGURE 3:**
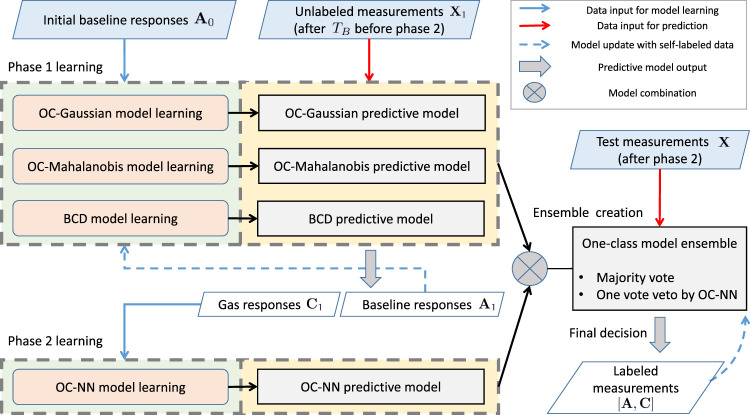
Schematic diagram of the ELBA algorithm. More detailed illustrations for the model learning phase 1 and 2 are in shown Figures 4A,B, respectively. (For interpretation of the references to color in this figure legend, please refer to the colour version of this article.)

Figure 3 depicts an overview of the proposed ELBA algorithm. Briefly, two model learning phases take place successively to create an ensemble composed of several base different models. Phase 1 deals with three one-class models targeting on baseline responses and phase 2 deals with a one-class model learning from gas responses. The models learned in both phases are combined as an ensemble for final prediction on unknown measurements. The two-phase learning process of the ELBA algorithm does not require training data of all possible target chemical analytes for initialization, and short-term drift is particularly addressed by performing online adaptive updates with self-labelled measurements during model learning or after the ensemble is complete.

**FIGURE 4:**
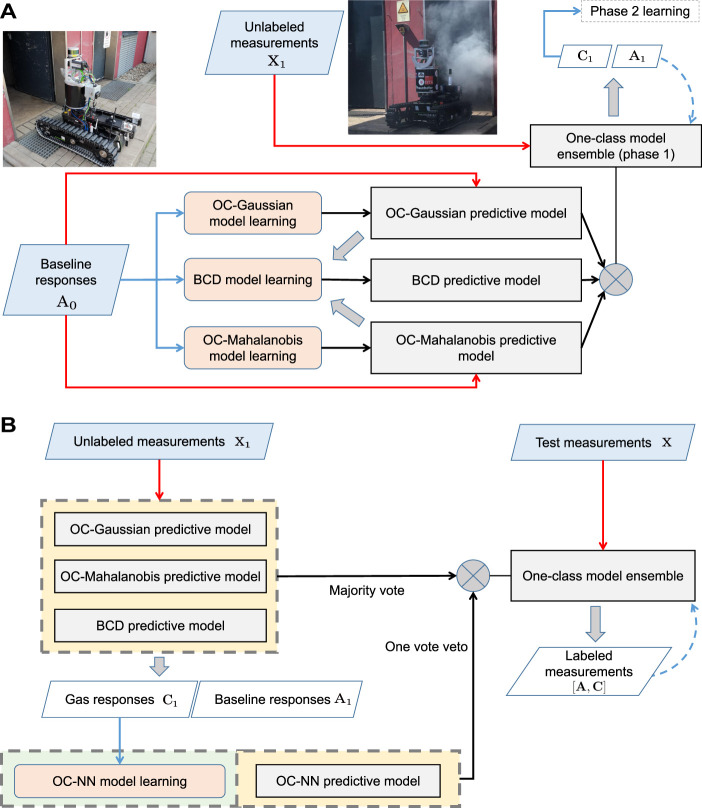
Phase 1 and phase 2 model learning of the ELBA algorithm. Please refer to *phase 1 learning*, *phase 2 learning* and *Ensemble creation* in Section 2.1 for corresponding details of this diagram. **(A)** In the phase 1 model learning, the e-nose is exposed to clean air to train the OC-Gaussian and the OC-Mahalanobis model, and the BCD model. Before phase 2 learning, these three models make predictions on new measurements with labels indicating them as baseline or gas responses. The self-labelled measurements are also used to update the models (when baseline responses are identified) or trigger phase 2 learning (when gas responses are detected). **(B)** In the phase 2 model learning, a One-Class Nearest Neighbour (OC-NN) model is trained from self-labeled non-air measurements with the models previously learned in phase 1. The models learned in phase 1 and phase 2 are combined to form the one-class ensemble for gas detection. The models can be future updated online with new self-labelled measurements.

The model learning process shown in Figure 3 is decomposed into three steps, namely *phase 1 learning*, *phase 2 learning* and *ensemble creation*. Below each step is described in order:

1. *Phase 1 learning* At the beginning of a gas sensing task, phase 1 model learning is performed first. In this phase, three one-class models are learned as descriptors of baseline responses, which are a One-Class Gaussian model (OC-Gaussian), a Mahalanobis-based One-Class model (OC-Mahalanobis), and a Bout-Count Detection (BCD) model. As shown in Figure 4A, the OC-Mahalanobis and the OC-Gaussian models are trained with baseline responses **A**_0_. In practice, the initial training data **A**_0_ can be prepared as follows: before the sensor array is deployed into the target field, the sensor array is ensured to interact with clean air for a period of time *T*_*B*_. During *T*_*B*_, the collected measurements are considered as the **A**_0_.

Once the OC-Gaussian and the OC-Mahalanobis model estimate the distribution of baseline responses from **A**_0_, they are used as predictive models to indicate the likelihoods of being clean air for new measurements. Based on these indices, the identities of the considered measurements are decided. The determinations are made with corresponding likelihood thresholds (one for the OC-Mahalanobis model and one for the OC-Gaussian model) that are learned from likelihood indices of **A**_0_.

In parallel, the bout-count detection model learns the baseline response pattern based on extracting latent information from the particular type of transient signals called bouts (Schmuker et al., 2016), which are the rising edges in the first derivatives of the sensor responses. The BCD model aims to catch the difference in the transient signals between baseline responses and gas responses. The BCD model aggregates the bout detection results from several signal sources under clean air conditions, which are assumed to be distinct from bouts induced by approaching gas exposures. The input signals of the BCD model are raw gas sensor responses, and the outputs of the OC-Mahalanobis and the OC-Gaussian models. These signals acquired in *T*_*B*_ are processed to decode underlying bouts, whose amplitudes are considered as a representative distribution of bouts in clean air conditions.

Before the phase 2 learning is conducted, new measurements are acquired as the gas sensing task is ongoing. The measurements acquired after phase 1 learning and before phase 2 learning are denoted as **X**_1_. Unknown measurements **X**_1_ will be processed by the previously learned three models. As a result, the recognized baseline responses **A**_1_ are used to update the baseline models, and the detection of gas responses **C**_1_ will induce phase 2 learning.

2. *Phase 2 learning* When the e-nose is exposed to an analyte for the first time, the corresponding measurements are detected as outliers by the three models learned in phase 1. Consequently, phase 2 model learning is triggered once an amount of gas responses are acquired. As shown in Figure 4B, the measurements **C**_1_, which are detected and labelled as gas responses, are used to train the One-Class Nearest Neighbour (OC-NN) model.

3. *Ensemble creation* Once both phase 1 and phase 2 learning are complete, the one-class models are fused to form the ensemble learning-based gas detection system. Specifically, the learned models are combined using the majority voting scheme, while the OC-NN model has one vote veto for determining baseline responses.

In the rest of gas sensing task, the built model ensemble makes predictions on the new test measurements, recognizing them as gas responses or baseline responses (i.e., performing gas detection). The labeled measurements can be used to retain the models online and to adapt to the drifted baseline offset.

### 2.2 The Base One-Class Classifiers

This section describes each base one-class model in detail, namely the One-Class Gaussian, One-Class Mahalanobis, and Bout-Detection models learned in phase 1 and the One-Class Nearest Neighbours learned in phase 2.

#### 2.2.1 One-Class Gaussian Model

The One-Class Gaussian model and the One-Class Mahalanobis model compensate each other in this ensemble learning-based approach. The considered one-class Gaussian treats each sensor response as independent variables, whereas the OC-Mahalanobis model assumes that the responses of each sensor are correlated. A typical one-class Gaussian classifier is a density-based model that assumes the data of the target class form a multivariate Gaussian distribution (Tax, 2002).

For a given *n* − dimensional measurement **r** as an input, its probability of belonging to the target class can be estimated with the probability density function (PDF) of a unimodal Gaussian distribution. Such a multivariate Gaussian model assumes that the cross-sensitivity of the MOX sensors is reflected by the covariance matrix of the variables. Since this assumption is already considered in the OC-Mahalanobis model with more robust covariance estimation, we propose a dedicated model for situations where this assumption does not hold. The baseline responses are modeled as a linear combination of several equally weighted single Gaussians (Tax, 2002), where the parameters of each Gaussian are learned from the initial sensor responses individually. This one-class Gaussian model yields the likelihood indicator of a test measurement, *s*_*GM*_, as follows:

$$s_{GM}\left(r_{i}\right)=\frac{1}{n}\overset{n}{\underset{j=1}{\sum }}\left(1-\frac{1}{n}\int_{0}^{r^{j}}P\left(r\right)dr\right)$$ (1a)

$$P\left(r^{j}\right)=\frac{1}{\sqrt{2\pi {\left(\alpha \cdot \sigma^{j}\right)}^{2}}}e^{-\frac{{\left(r^{j}-\mu^{j}\right)}^{2}}{2{\left(\alpha \cdot \sigma^{j}\right)}^{2}}}$$ (1b)

where *μ* ^*j*^ is the mean of *j*th sensor responses of all measurements in **A**, and *P*(**r** ^*j*^) is the PDF of the distribution estimated by the *j*th sensor response of the baseline training samples. *α* is a free parameter that scales the estimated variance of the each sensor responses. *α* ⋅ *σ* ^*j*^ determines the boundaries of the Gaussian model for the *j*th sensor.

The output of the OC-Gaussian modle, *s*_*GM*_, is further processed by the BCD model as one of the input signals (along with the output of the OC-Mahalanobi model and raw sensor responses). The prediction by the OC-Gaussian model depends on a predefined decision function with a learned threshold, such that the test measurement is clean air if *s*_*GM*_ < = *λ*_*GM*_. Given the learned OC-Gaussian model and the set of baseline responses **A**, we let

$$\lambda_{GM}=\underset{r_{i}\in A}{max}\left\{s_{GM}\left(r_{i}\right)\right\}+k_{GM}\cdot \sigma_{A}$$ (2)

where *k*_*GM*_ is a free parameter empirically set to 3.

#### 2.2.2 The Mahalanobi Distance-Based One-Class Model

The Mahalanobis-based one-class model (OC-Mahalanobis) is selected to learn the pattern of baseline responses, taking the correlation between the sensors (cross-sensitivity) into consideration. The OC-Mahalanobis is a distance criterion approach that distinguishes a measurement from baseline responses based on their similarity expressed by the Mahalanobis distance. The Mahalanobis distance is a distance metric between a data point and a distribution, which has been considered as an indicator of class separability between chemical analytes, for example, in (Bennetts et al., 2014). Here we use *d*_*MD*_ as a metric to quantify the degree of similarity between an observed measurement and the baseline response distribution. The output of the model *d*_*MD*_ will be used an index to reflect the likelihood of being air for the input, i.e., an instantaneous measurement.

The calculation of the Mahalanobis distance requires to estimate the covariance of the underlying distribution. However, using the covariance maximum likelihood estimate could be sensitive to possible outliers in the data set. To improve the robustness of Mahalanobis distance, the Minimum Covariance Determinant estimator (MCD) has been applied as a robust estimator of covariance to make the estimation resistant to outliers (Rousseeuw, 1984), and therefore the associated MCD-based Mahalanobis distances can accurately reflect the separability against the inliers.

Given a set of representative measurements of baseline responses, **A**, the robust MCD-based Mahalanobis distance between a measurement **r** and **A** is given by

$$d_{MD}\left(r,A\right)=\sqrt{{\left(r_{i}-{\hat{\mu }}_{MCD}\right)}^{T}{\hat{\Sigma }}_{MCD}\left(r_{i}-{\hat{\mu }}_{MCD}\right)}$$ (3)

where ${\hat{\mu }}_{MCD}$ is the MCD estimate of the mean of **A**, and ${\hat{\Sigma }}_{MCD}$ is the MCD covariance estimate, both of which rely on an off-the-shelf Scikit-Learn implementation (Pedregosa et al., 2011).

Similar to the previous OC-Gaussian model, the output of the OC-Mahalanobis model, *d*_*MD*_, is fed into the Bout-Count Detection (BCD) model. The corresponding pipeline structure will be described in Section 2.2.3. Besides, the obtained *d*_*MD*_ is also used to derive a measure for being an outlier with the Mahalanobis-based one-class classifier.

This one-class predictive model requires to set a threshold *λ*_*MD*_ to represent the decision boundary of the baseline responses. The decision function of the OC-Mahalanobis model considers the test measurement as clean air if its *d*_*MD*_ is greater than this threshold, i.e., *d*_*MD*_ < *λ*_*MD*_ (Nader et al., 2014). The value of *λ*_*MD*_ is determined by Eq. 4, as follows:

$$\lambda_{MD}=\underset{r_{i}\in A}{max}\left\{d_{MD}\left(r_{i},A\right)\right\}+k_{MD}\cdot \sigma_{A}$$ (4)

where *σ*_**B**_ is the standard deviation of the pairwise Mahalanobis distances within the group of baseline responses ({*d*_*MD*_(**r**_*i*_, **A**)∣**r**_*i*_ ∈ **A**}). The purpose of adding the term *k*_*MD*_ ⋅ *σ*_**B**_ is to extend the boundary of the baseline responses distribution to account for possible sensor drift. The parameter *k*_*MD*_ scales with the variance of the distribution of baseline responses. *k*_*MD*_ is a free parameter that is set empirically in the range from 3 to 5. The advantage of using a one-class model is that it only requires exposing the sensor to clean air for a predefined period.

#### 2.2.3 Bout-Count Detection Model

Significant changes caused by gas exposures are reflected in the transient signals of sensor responses. Different types of features based on transient signals have been proposed to extract relevant information regarding an analyte (Gutierrez-Osuna et al., 1999; Carmel et al., 2003; Muezzinoglu et al., 2009). Recently, it has been found that the number and frequency of a transient feature called bout are highly associated with the distance towards a gas source (Schmuker et al., 2016; Burgués and Marco, 2020b). The correlation between bouts and the distance to a gas source implies that bouts have the potential to indicate gas detection events. In the previous related works that utilize bouts to indicate gas source distances, bout is referred to as a transient feature defined as the rising edges in the considered sensor response or the first derivatives of the sensor response. Compared the instantaneous sensor responses processed by the OC-Gaussian and the OC-Mahalanobis models, the derivative-based bout has the advantage of making small changes more noticeable. In addition, it has a potential in detecting peaks in sensor response that are superimposed on and obscured by stronger but broader background (O’Haver, 1997). The latter property might be important for gas detection with mixtures, although target analytes in the form of mixture are not in the scope of this work.

In the proposed Bout-Count Detection (BCD) model, we further integrate the ensemble learning technique stacking to increase the diversity of the signal sources from which bouts are extracted: in addition to the raw sensor responses, the outputs of the OC-Gaussian and the OC-Mahalanobis models (i.e., *s*_*GM*_ and *d*_*MD*_, respectively), are considered as stacked signal sources for bout count detection. This means, both bouts from the sensor responses but also from the stacked signals are detected and analyzed to determine gas detection events. We assume that use stacking can enhance model diversity because the signals *s*_*GM*_ and *d*_*MD*_ are calculated by different predictive models, and both of them are supposed to be more comprehensive than a single raw sensor response.

**FIGURE 5:**
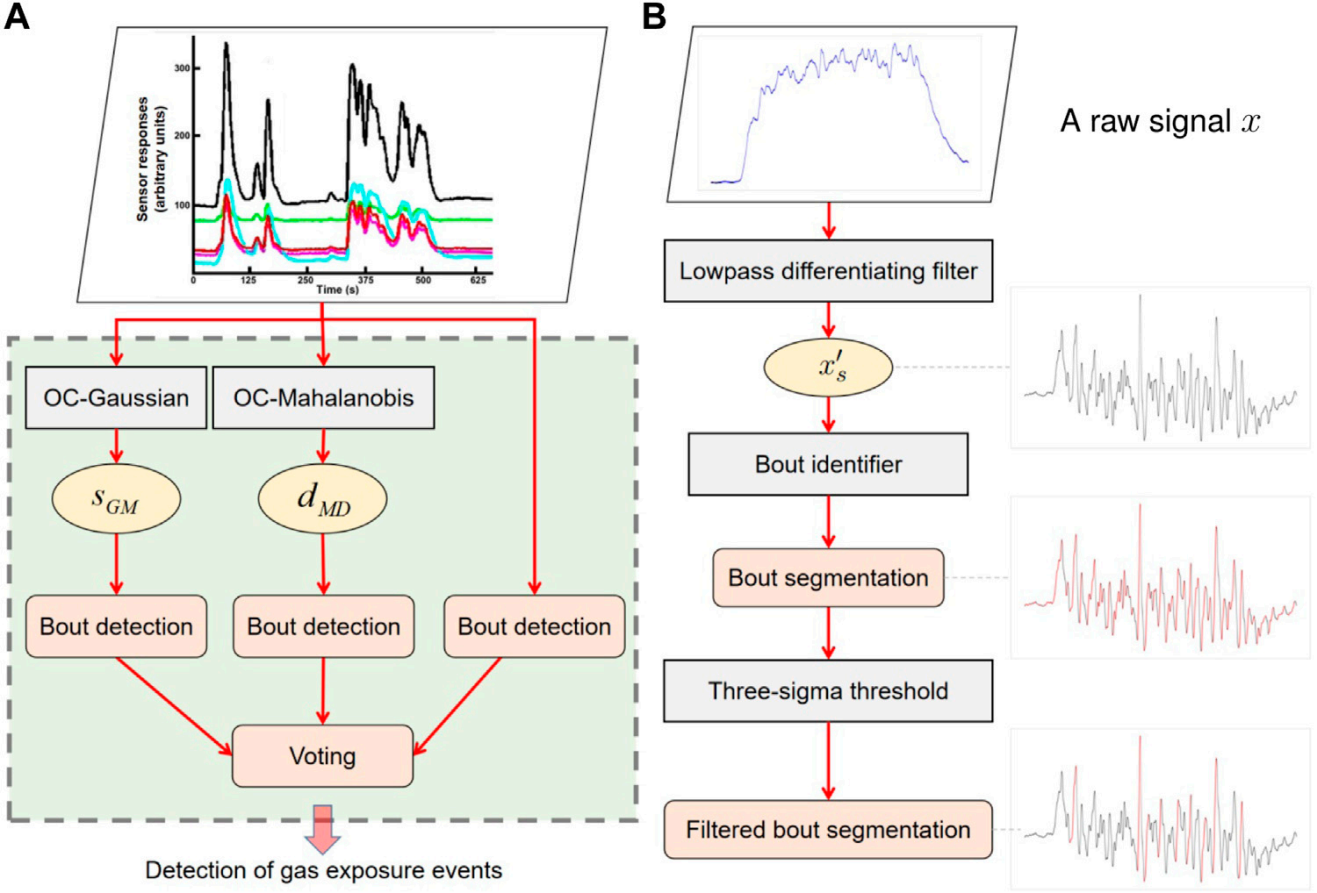
Diagram of the Bout-Count Detection (BCD) model and the algorithm used for each base bout detection model. **(A)** Pipeline of the BCD model. **(B)** Diagram of the bout detection algorithm used in the BCD model. A raw signal will be filtered to remove high-frequency noise first. From the filtered signal *x*_*s*_, bout segments that fit the definition $x_{s}^{\prime \prime }>0$ in are be identified, which are indicated by the red curves. The bouts passing a three-sigma threshold are further selected as they are considered to be useful for gas detection. The amplitudes of the detected and filtered bouts are for illustration purpose.

Figure 5A depicts the learning pipeline of the Bout-Count Detection Model. As is shown, the structure of the BCD model is stacked, where the outputs of the OC-Gaussian and the OC-Mahalanobis model are used as input features to respective bout detectors. According to previous studies, a stacked ensemble tends to provide improved performance than using the considered models individually or using the single best model (Wolpert, 1992; Smyth and Wolpert, 1999).

The final prediction of the BCD model is made from a voted ensemble of the base bout detection models. The bout extraction algorithm used in each base bout detection model is adopted from (Schmuker et al., 2016). As illustrated in Figure 5B, the detection of the bouts consists of the following consequent steps:

1) The derivative $x_{s}^{\prime }$ of the smoothed signal *x*_*s*_ is computed. First, high-frequency noise in the raw signal *x* is smoothed using convolution with a Gaussian kernel (low-pass filtering).
2) Bouts of rising amplitude are searched in the derivative of the smoothed signal. A bout is found when $x_{s}^{\prime \prime }>0$ , and it terminates as $x_{s}^{\prime \prime }$ turns back to 0. The bout amplitude is defined as the difference between the values of $x_{s}^{\prime }$ at the beginning and the end of the respective bout. Here, the use of the second derivative is similar to the discrete Laplace operator for edge detection in image analysis (Mlsna and Rodríguez, 2009). Using $x_{s}^{\prime \prime }$ is supposed to be more sensitive to significant small changes as well as noise. Since the signal has been smoothed in the previous step, the sensitivity to noise should not be an issue. Otherwise, using the first derivative directly is an option for this step (Burgués and Marco, 2020b).
3) The bouts are further processed to exclude those of low amplitude. According to (Schmuker et al., 2016) and (Burgués and Marco, 2020b), the bouts of low amplitude are considered to be produced by noise and therefore are filtered out by thresholding. For simplicity, the thresholding is also adopted to the BCD model for the final bout declaration. As a trade-off between detection sensitivity and false-positive reduction, the three-sigma criterion is utilized, which has been empirically demonstrated to be sufficient for extracting informative bouts in (Schmuker et al., 2016). Relying on the assumption that the distribution of bout amplitudes detected from baseline responses can be approximated by a Gaussian distribution, a fixed amplitude threshold can be set with the three-sigma rule as follows:

$$\theta_{BD}=\mu_{\mathrm{BD}}+3\cdot \sigma_{\mathrm{BD}}$$ (5)

where *μ*_**BD**_ and *σ*_**BD**_ are the sampled mean and standard deviation of the bout amplitude distribution whose bouts are extracted from baseline responses. These bouts are supposed to have smaller amplitudes than bouts extracted from gas responses, and such a difference in amplitude is the key to identify baseline responses.

#### 2.2.4 One-Class Nearest Neighbour Classifier

The One-Class Nearest Neighbour classifier (OC-NN) learned on gas responses contributes to the ensemble model. One of the key factors attributing to the effectiveness of ensemble learning is ensemble diversity (Zhou, 2012; Löfström, 2015). Diversity of models can be achieved by combining models of different working principles or/and allowing the models to learn from different training data (Zhou, 2012). Both strategies are utilized here. The OC-NN model is trained with self-labelled gas responses, which differs from the OC-Mahalanobis and the OC-Gaussian models that are learned from baseline responses. In addition, the basic principle of the OC-NN model is based on a different assumption from that of the OC-Mahalanobis and the OC-Gaussian models. The OC-NN model assumes that measurements within the target class are closer to each other in the feature space than to outliers. The training of the OC-NN model takes place in phase 2 model learning, which requires the one-class models learned in phase 1 recognize gas responses as the training data. Once the OC-Mahalanobis, the OC-Gaussian, and the BCD models are constructed, phase 1 model learning is complete. Gas detection can be performed to recognize the measurements that significantly deviate from clean air using the ensemble of these three one-class classifiers. The detected measurements, which can be assumed to be caused by exposure to chemical analytes, trigger the subsequent phase 2 model learning. In phase 2, a One-Class Nearest Neighbour (OC-NN) model of gas responses is learned during exposure of the e-nose. The OC-NN used in this work is based on the two-layer-neighbourhood one-class model proposed in (Khan and Ahmad, 2018).

The measurements detected by the models of the OC-Mahalanobis and the OC-Gaussian are self-labelled as gas responses, as an opposite class of clean air. These measurements are used as the target class to train the OC-NN model. We assume that the inter-class distances between gas responses of different analytes are comparatively smaller than the inter-class distance between gas responses (of an analyte) and baseline responses. According to our previous study (Fan et al., 2019a), this assumption holds, which is the basis of the OC-NN model to perform gas detection on multiple analytes as long as the e-nose is sensitive to them.

The OC-NN model used in the ELBA algorithm is coupled with a binary probabilistic classifier, which allows to produce a prediction score over the class of clean air, given a test measurement. The prediction scores are estimated using Platt scaling (Niculescu-Mizil and Caruana, 2005), which is implemented by fitting a logistic regression with the initial baseline responses **A** and self-labelled gas responses. The input of ad hoc logistic regression is a distance-based feature extracted with the aforementioned two-layer-neighbourhood structure, which is expected to better characterize the boundary between the two classes.

The learning procedure of the OC-NN model is as follows:

1) The non-air measurements detected by the OC-Gaussian and the OC-Mahaalanobis models are taken as the representative data of the gas responses, which is also the target class of the OC-NN model.
2) The OC-NN model is used to transform the instantaneous sensor response of a test measurement into a feature *d*_*NN*_ using Algorithm 1. The value of *d*_*NN*_ reflects how much a test measurement deviates from the class of gas responses. The higher value *d*_*NN*_ is, the greater chance that the corresponding test measurement is different from known gas responses.
3) Measurements of both the target class **X** and the initial baseline **A** are processed by Algorithm 1, resulting in a training set of two classes. A binary logistic regressor *LR* is trained with this training set, taking *d*_*NN*_ of a test measurement as input and outputting a prediction score *s*_*NN*_. The value of *s*_*NN*_ reflects how much a test measurement deviates from the class of gas responses. The higher value *s*_*NN*_ is, the greater chance that the corresponding test measurement is distant from known gas responses.
4) In order to obtain a decision boundary, the *s*_*NN*_ scores of baseline responses in **A** are calculated to find their mean *μ*_*NN*_ and standard deviation *σ*_*NN*_. The decision threshold of OC-NN is defined as *λ*_*NN*_ = *μ*_*NN*_ − 3*σ*_*NN*_. Test measurements are identified as a baseline response if *s*_*NN*_ > *λ*_*NN*_.

Algorithm 1

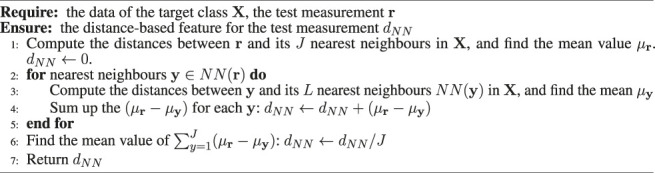

Once the OC-NN model is learned, the next step is to create the model ensemble for gas detection. For a test measurement, its label (gas response or baseline response) is determined on the following principles:

• The majority vote of the BCD, the OC-Mahalanobis, the OC-Gaussian, and the OC-NN model first determines if a test measurement is a gas response or not.
• The prediction of the OC-NN model is assigned to have “one vote veto”: a test measurement will not be recognized as a gas response if *s*_*NN*_ > *λ*_*NN*_.

The reason for allowing the OC-NN to have a “one vote veto” is to reduce false alarm (i.e. identifying clean air measurements as gas responses) (Khan and Ahmad, 2018). suggest that the original two-layer-neighbourhood model adopted by OC-NN might accept more non-targets as members of the target class. As a possible consequence, the OC-NN could have a tendency to be conservative in declaring gas detection (given that its target class is gas responses). This means, when the OC-NN detect gas responses, it is not likely to a false alarm.

### 2.3 Measures of Diversity in Ensemble Learning

The ELBA algorithm consists of several diverse models. As is known, diversity among the base models is deemed to be an important factor in ensemble construction (Zenobi and Cunningham, 2001; Kuncheva and Whitaker, 2003; Tang et al., 2006). In the proposed ensemble, the diversity among the individual models is generated by using different working principles or/and training data. For evaluation purpose, we will use several diversity measures to quantify the difference in diversity between using an ensemble and using the base models individually.

In the scope of the proposed approach, diversity is referred to as the extent to which the outputs of the models agree on gas detection given the same sensor responses. The degree of agreement among prediction results can be quantified with diversity measures. Existing methods are categorized into pairwise and non-pairwise diversity measures (Kuncheva and Whitaker, 2003). The pairwise measures quantify diversity between two models based on their predictions. To quantify the overall diversity for an ensemble requires calculating the average of the pairwise diversity for all combinations of two distinct models. Non-pairwise measures allow an assessment of the overall diversity of more than two models directly.

In this paper, three non-pairwise measures are adopted: Fleiss’ Kappa, entropy measure, and Kohavi-Wolpert variance. In the calculation of these diversity measures, the binary outputs (clean air or gas response) of the predictive models are considered.

#### 2.3.1 Kohavi-Wolpert Variance

(Kohavi and Wolpert, 1996) introduced the following expression to quantify the variability of the predicted class label given input data *r*_*i*_ using a model.

$$var\left(r_{i}\right)=\frac{1}{2}\left[1-\left(P{\left(\text{clean air}|r_{i}\right)}^{2}+P{\left(\text{gas response}|r_{i}\right)}^{2}\right)\right]$$ (6)

By averaging over the variance of each individual model, the Kohavi-Wolpert variance that reflects the overall diversity is defined as:

$$KW=\frac{1}{nM^{2}}\overset{n}{\underset{i=1}{\sum }}\overset{K}{\underset{j=1}{\prod }}m\left(r_{i},j\right)$$ (7)

where *M* is the number of considered models and *K* denotes the number of possible labels. The term *m*(*r*_*i*_, *j*) denotes the number of models that assign the measurement *r*_*i*_ with label *j*. In this way, *m*(*r*_*i*_, clean air/gas response)/*M* approximates the probability *P*(gas response∣*r*_*i*_) or *P*(clean air∣*r*_*i*_).

Identical models will result in *KW* = 0, and higher Kohavi-Wolpert variance indicates an increased degree of diversity.

#### 2.3.2 Entropy Measure

The use of entropy to measure ensemble diversity was first introduced by Cunningham and Carney (Cunningham and Carney, 2000). In this work, we take Kuncheva and Whitaker’s definition in (Kuncheva and Whitaker, 2003), and adopt it as follows

$$H=\frac{1}{n}\overset{n}{\underset{i=1}{\sum }}\frac{1}{M-\left(M+1\right)/2}\text{min}\left\{m\left(r_{i},\text{gas response}\right),m\left(r_{i},\text{clean air}\right)\right\}$$ (8)

where *n*, *M* and *m*(*r*_*i*_, *j*) share the same denotations as in Eq. 7.

The entropy measure *H* is between 0 and 1. *H* = 0 is observed when the considered models give identical predictions, while *H* = 1 indicates that the models are perfectly diverse.

#### 2.3.3 Fleiss’ Kappa

Fleiss’s Kappa *K*_*F*_ is also referred to as the interrater agreement. It is a statistical measure of the reliability of agreement between a number of raters (Fleiss et al., 1981). In the context of this paper, a rater would be a predictive model that labels input data as clean air or gas responses. Intuitively, *K*_*F*_ calculates the degree of agreement between models in prediction occurring by chance. It is defined as follows:

$$K_{F}=\frac{P_{o}-P_{e}}{1-P_{e}}$$ (9)

where *P*_*o*_ gives the degree of agreement among models that is actually observed and *P*_*e*_ corresponds to the degree of agreement that is attainable by chance (Randolph, 2005). Again, the notations are the same as in Eqs. 7 and 8: *n* denotes the total number of measurements, *M* denotes the number of considered models, *K* denotes the number of possible labels (in this case, *K* = 2), and *m*(*r*_*i*_, *j*) denotes the number of models that assign the measurement *r*_*i*_ with label *j*. *P*_*o*_ is given by

$$P_{o}=\frac{1}{nM\left(M-1\right)}\left(\overset{n}{\underset{i=1}{\sum }}\overset{K}{\underset{j=1}{\sum }}m{\left(r_{i},j\right)}^{2}-nM\right)$$ (10)

and *P*_*e*_ is given by

$$P_{e}=\overset{K}{\underset{j=1}{\sum }}{\left(\frac{1}{nM}\overset{n}{\underset{i=1}{\sum }}m\left(r_{i},j\right)\right)}^{2}$$ (11)

*K*_*F*_ ∈ (0, 1] between 0 and 1 indicate that the degree of agreement is achieved above chance, and *K*_*F*_ = 0 indicates a level of agreement that could be expected by chance. *K*_*F*_ ∈ (0, −1] indicates that there is no agreement among the models (other than what would be expected by chance). The smaller the *K*_*F*_ is, the more diverse the models are.

## 3 Data Set and Experimental Set-Up

**FIGURE 6:**
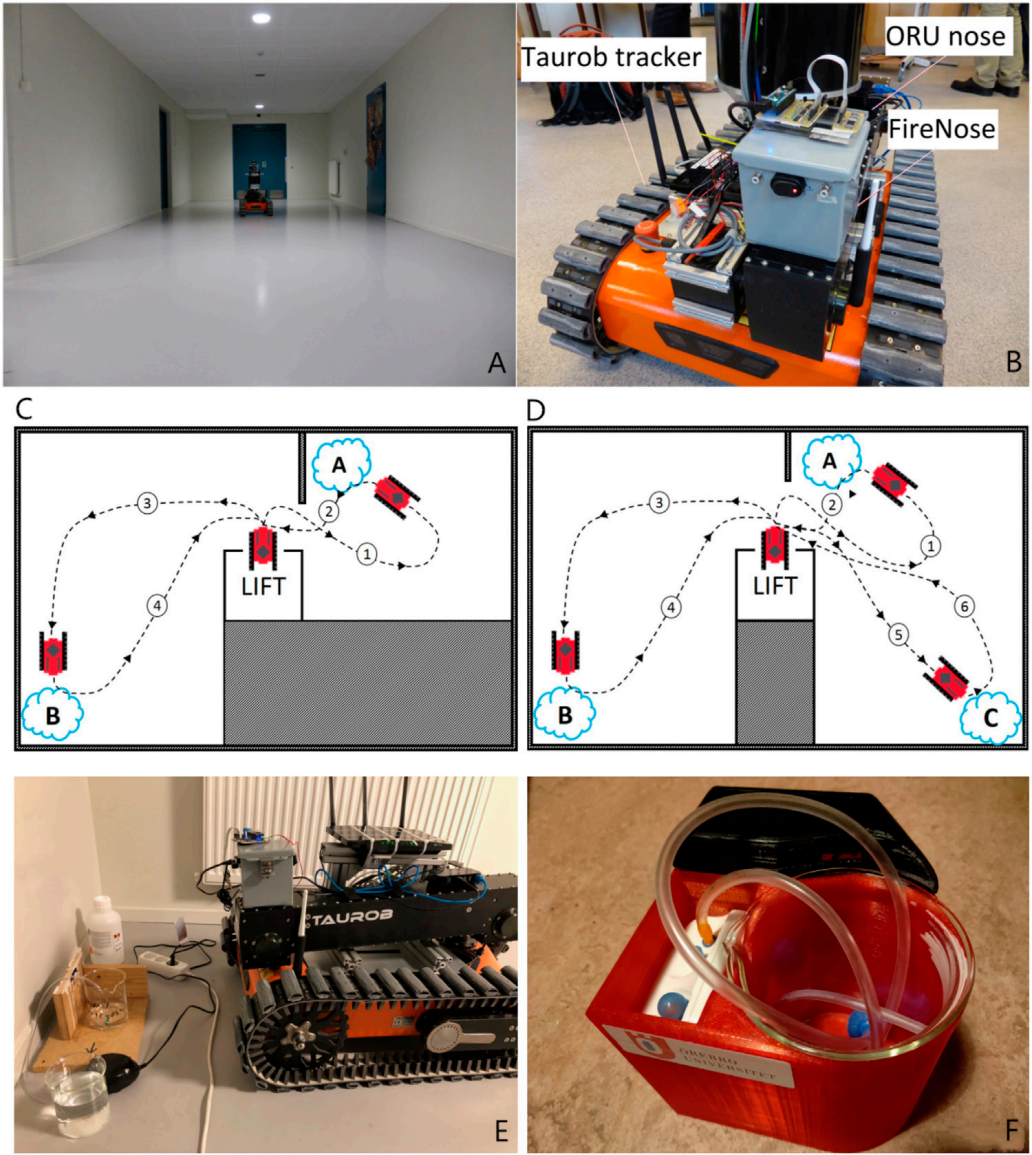
The experimental setup. **(A)** The basement environment of the field experiment reported in this paper. **(B)** A Taurob tracker is used as the platform to carry the gas sensing payload, namely the FireNose and ORU nose. **(C)** and **(D)** Schematic drawings of the 2-source trials and the 3-source trial, respectively. **(E)** A simple gas source set-up used in the early trials of the experiment. **(F)** Standardized gas source used in later trials, which that consists of a fan and an air pump bubbler covered by a 3D printed case.

In order to evaluate the proposed algorithmic set-up of the gas sensing system, it was implemented on a search and rescue robot. The robot has been tested in a basement of a public building (Figure 6A). In Section 4, we present the results of two experiment trials conducted in this basement environment.

### 3.1 Robotic Platform

The robotic platform used is a Taurob tracker (Biegl et al., 2014) (shown in Figure 6B), which is built to help CBRN (Chemical, Biological, Radiological and Nuclear) first responders, EOD (explosive ordnance disposal) teams, fire-fighters and search & rescue teams to gain first-hand information for emergency response. The Taurob tracker can be equipped with a novel 3D radar camera, a 3D laser scanning sensor and a thermal camera that allow for robust mapping and navigation under low visibility conditions (Fritsche et al., 2018). The unknown and uncontrolled sensing conditions assumed in this work are possibly shared with the target environments of Taurob robots. However, the capability of the robot platform operating in harsh environments is out of the scope of this paper.

### 3.2 Electronic Noses

As shown in Figure 6B, the robotic platform was equipped with two different gas sensor units, namely a commercial MOX sensor array (ORU nose) and a prototype FireNose composed of several gas sensors developed by (Wei et al., 2016).

The FireNose incorporates three MOX sensors coated with tin oxide (SnO_2_), tungsten oxide (WO_3_) and nickel oxide (NiO), respectively. During the sampling process, the ambient air is drawn into the sensor chamber through a pipe by a micro air pump. An airflow rate sensor is employed to monitor the volumetric flow rate, which is used as a reference measure to control the pump speed. The measurements are filtered by a physical filter to eliminate smoke particles and further moved into a measurement chamber to interact with the MOX gas sensors. The raw sensor responses are generated at 100 Hz, and they are pre-processed by an on-board micro controller in real-time with digital filters, peak detection, and fast Fourier transform. After these signal processing procedures, the responses are sampled at 2 Hz as the sensor output.

The ORU nose includes six commercial MOX sensors fabricated by SGX Sensortech, namely, MICS-2614, MICS-5524, MICS-5914, MICS-2714 and two MICS-4514. According to the manufacturer, they are highly sensitive to volatile organic compounds. Contrary to FireNose, the sensors are not housed inside a chamber. Instead, they are directly exposed to the environment in what is commonly referred to as an open sampling configuration. The responses are sampled at 2 Hz. The ELBA approach uses responses of the MICS-2614 (referred to as s 1), the MICS-5914 (referred to as s 3) and one of the MICS-4514 (referred to as s 6) as the input. The selection of the sensors is as same as the setup used in (Xing et al., 2019). This setup was confirmed to be sensitive to ethanol, propanol and acetone and sufficient to support an unsupervised gas discrimination task, but it is not optimized for a gas detection purpose.

### 3.3 Experimental Set-Up

Two experiment trials of different set-ups were conducted to validate the presented approach (see Table 1). The experimental environment is a basement with a narrow corridor connecting two rooms, which is a typical open environment. The large space allows gas exposures to be separated temporally and spatially, so that gases can distribute with minimal interference with each other. Alternative setup that allows gases from different sources to evolve into a mixture, for example studied in (Maho et al., 2021), will be considered in future work.

**TABLE 1:** Summary of the set-ups of the field experiments for the ELBA algorithm evaluation.

Experimental Trial	Gas Sources		Distance Between the Sources	Time Interval Between exposures^a^
	Source 1	Source 2		
Exp. 2-source	Ethanol	propanol	14.24 m	7 min 44 s
Exp. 3-source	$\left\{\begin{array}{c} \text{ethanol }\left(\text{1a}\right)\\ \text{ethanol }\left(\text{1b}\right) \end{array}\right.$	Acetone	$\left\{\begin{array}{c} 1\text{a Vs. }1\text{b: }1.98\text{m}\\ 1\text{a Vs. }2\text{: }6.91\text{m}\\ 1\text{b Vs. }2\text{: }8.36\text{m} \end{array}\right.$	$\left\{\begin{array}{c} 1\text{a to }1\text{b:}17\text{ min }45\text{s}\\ 1\text{b to }2\text{:}5\text{ min }40\text{s} \end{array}\right.$

a A time interval between exposures is counted on the condition that the robot is at least 3 m away from the closest gas source.

We made no explicit effort to regulate the environmental conditions, such as temperature, airflow, and pressure. Since the regulations did not allow the release of toxic gases into the environment, we use three kinds of commercially available liquids as analytes: ethanol (95% pure), 1-propanol (99.5% pure) and acetone (100% pure). In a previous study, FireNose showed sensitivity to these chemical compounds (Xing et al., 2017).

In the Exp. 2-source trial, two sources were placed at corners of two basement rooms, marked on Figure 6C as “A” (first gas source) and “B” (second gas source). Each gas source comprised a bubbler system, in which propanol and ethanol VOCs (in liquid form) were bubbled to facilitate their evaporation into the environment. A small pump was used to generate a constant air supply at each source (see Figure 6E for example). In the later trials, a standardized gas releasing device was used (Figure 6F). Before the start of each experimental trial, the sensor arrays were allowed to pre-heat for a period between 10 and 30 min, which is a standard warm-up time for MOX sensors. A trial began with the robot transported to the basement via a lift, which was treated as a clean area free from any chemical analytes released in the experiments. The robot stayed in this area to obtain baseline readings at the beginning of the experiment and after each gas exposure. A period of 5 min, i.e., *T*_*B*_ (Section 2), was allowed for the gas sensors to be exposed to clean air and therefore to generate a amount of stable baseline readings. The robot then travelled towards the first gas source “A” (path ), where the robot stayed stationary for 2–3 min (at approx. 1 m distance) to sample measurements before travelling back to the lift area to possibly collect further baseline readings (path ). Then the robot travelled to the second gas source “B” for the detection of another type of gas (path ). Again, the robot remained stationary for 2–3 min before returning to the ‘clean area’ (path ). The Exp. 3-source trial was set with three sources, namely two ethanol sources and an acetone source, respectively. In this trial, the robot approached the first and the second ethanol source one at a time and then moved on to the acetone source marked on Figure 6D as “C” following path. Similar to the 2-source trials, the robot returned to the clean air dominated area between each exposure.

## 4 Results

The ELBA algorithm is evaluated with in-field experiments described in the last section. As a negative effects of uncontrolled environmental conditions, the three-phase sampling strategy was inapplicable. Also, the baseline does not reach its initial value after recovery. As mentioned in Section 1, these issues pose challenges for a gas detection task.

The rest of this section presents a twofold evaluation of the one-class models with real-world experimental trials. First, the bout-detection based model is assessed in terms of its detection behaviours and ensemble diversity. Second, we perform a similar evaluation for the one-class models using direct instantaneous measurements. In both evaluations, we have observed differences among the one-class models in their reactions to sensor response changes and improvements in ensemble diversity from using the models individually to using them as an ensemble. The observations tentatively support the proposed ensemble learning scheme and the choice of the one-class models.

### 4.1 Evaluation of the Bout Detection-Based Model

**FIGURE 7:**
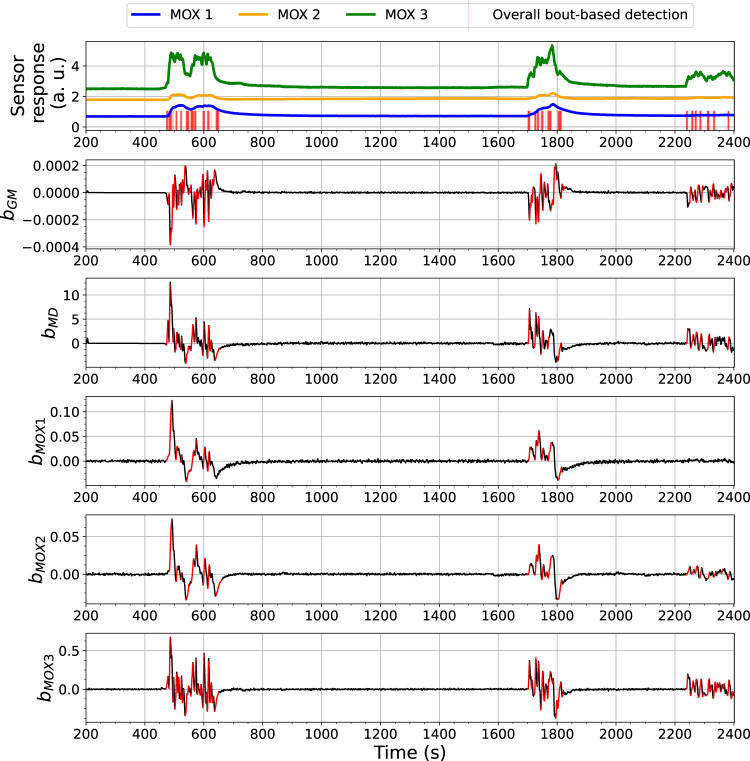
Bout analysis and the prediction result of the Bout-Count Detection (BCD) model using FireNose on the Exp. 3-source trial. The overall bout detection made by the BCD model is visualized in the top subfigure, where time steps within the duration of a found bout are marked with red vertical lines. The detected and filtered bouts in each signal are highlighted in red in the rest subfigures. A zoomed-in snapshot of this prediction result, shown in Figure 9, will illustrates the association between the overall bout detection and the individual bout detection on each signal.

**FIGURE 8:**
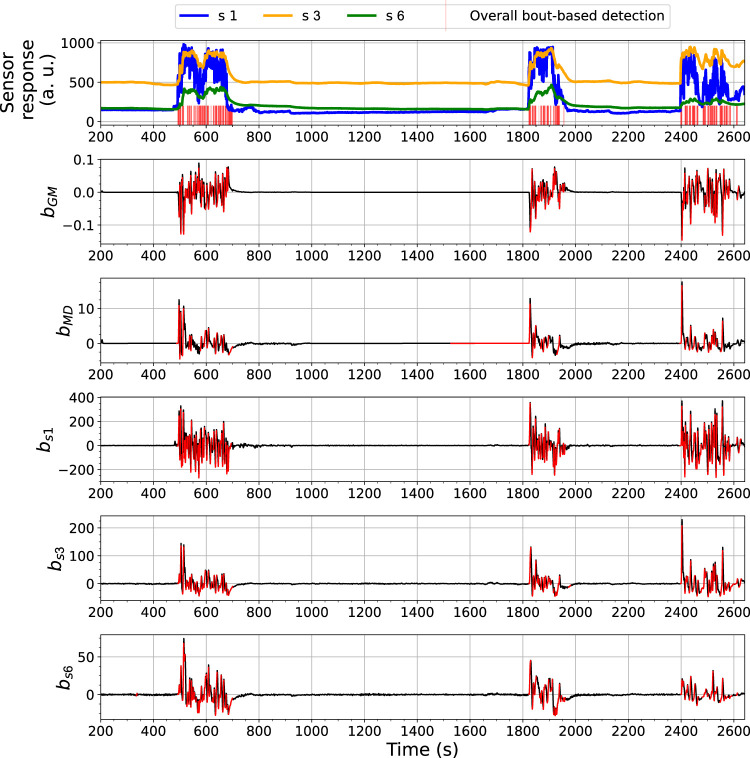
Bout analysis and the prediction result of the Bout-Count Detection (BCD) model using ORU nose on the Exp. 3-source trial. The overall bout detection made by the BCD model is visualized in the top subfigure, where time steps within the duration of a found bout are marked with red vertical lines. The detected and filtered bouts in each signal are highlighted in red in the rest subfigures.

The BCD model detects changes related to gas exposures with an analysis of bout-based features. Figures 7, 8 show the overall bout detection results in Exp. 3-source (the details of the data set can be found in Section 3). Figure 7 corresponds to the data set collected with FireNose and Figure 7 corresponds to the data set collected with ORU nose. From the time series of the sensor responses in both figures, one can observe that the e-nose using ELBA was exposed to gas three times, when the robot paused in front of two ethanol and one acetone sources, respectively. The overall output of the BCD model, i.e. the indications of gas detection events, comes from an ensemble prediction based on five individual bout detection results from *b*_*GM*_, *b*_*MD*_, *b*_*MOX1*_, *b*_*MOX2*_ and *b*_*MOX3*_. Blue impulses in the time series of the sensor responses visualize the time points that indicates the detected bouts.

**FIGURE 9:**
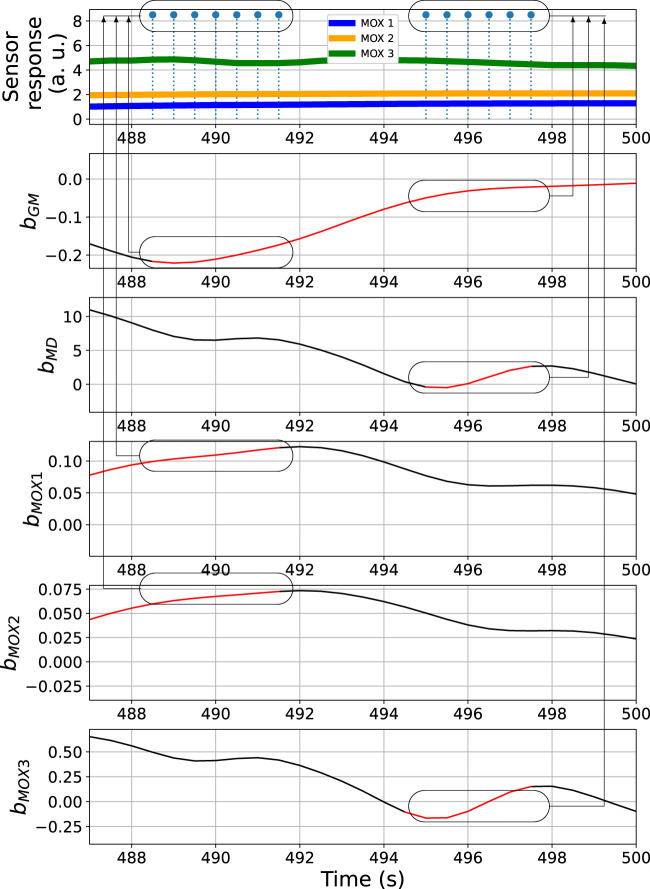
Bout analysis and prediction result of the period from 487 to 500 s of the time series shown in Figure 7. The ovals in the top subplot indicate the overall bout detection results, while the ovals in the other subplots correspond to the detected bouts contributed from each signal source.

From the overall bout detection results in Figures 7, 8, we can observe that these impulses approximately overlap with the duration of the three gas exposures in both data sets. Note that merely using bout detection of one single source might not be sufficient to catch all significant changes of the sensor responses. For example, from Figure 7 one can notice that, during the third gas exposure, the bouts detected from the sensor signal *MOX*1 are of small amplitudes compared with the bouts from the same source detected in the first and second gas exposure. While the models based on *b*_*MOX2*_, *b*_*MOX3*_, *b*_*MD*_ or *b*_*GM*_ exhibit significant bouts that exceed corresponding thresholds, the model based on *b*_*MOX1*_ does not contribute to the detection of the third gas exposure. The differences in the bout detection behaviours reflect diversity among the models using different signals. In the BCD model, this diversity is exploited by using a voting ensemble (previously illustrated in Figure 5A). Figure 9 provides a close look at how the voting ensemble makes the final prediction based on the bout analysis of each signal source. In the shown period from 487 to 500 s, there are two separate segments of blue impulses, which correspond to the overall bout detection results determined by the BCD model. In the first segment spotted, the signal *b*_*GM*_, *b*_*MOX1*_, and *b*_*MOX2*_ (3 out of 5 votes) contributed to the overall bout detection. In the second segment that occurred a few seconds later, it is the signal *b*_*GM*_, *b*_*MD*_, and *b*_*MOX3*_ that contribute the wining votes instead.

**FIGURE 10:**
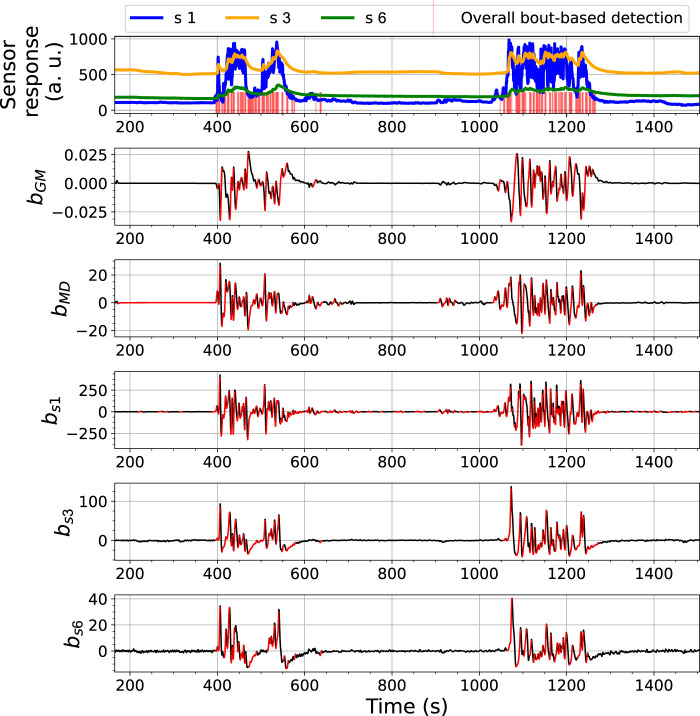
Bout analysis and the prediction result of the Bout-Count Detection (BCD) model using ORU nose on the Exp. 2-source trial. The overall bout detection made by the BCD model is visualized in the top subfigure, where time steps within the duration of a found bout are marked with red vertical lines. The detected and filtered bouts in each signal are highlighted in red in the rest subfigures.

The performance of the BCD model on the Exp. 2-source trial, e.g., as shown in Figure 10, is similar to what we have observed from the 3-source trial. In this shown case, approximately at *t* = 600 s, there was an signal increase in *s* 1, which is highly likely to correspond to a potential gas exposure. This change was caught by *b*_*GM*_, *b*_*MD*_ and *b*_*s*1_ but not *b*_*s*2_ or *b*_*s*3_, demonstrating the benefit of using an ensemble for the detection.

The diversity among the base models is of interest since it is a key factor of ensemble learning-based approaches in general. In this work, diversity is quantified with the measures described in Section 2.3, namely Fleiss’ Kappa, entropy measure and Kohavi-Wolpert variance. We thus set up three base model combinations and calculated the diversity measures for the three selected combinations. The first combination includes models that detect bouts from raw sensor signals, or in other words, based on *b*_*MOX1*_, *b*_*MOX2*_, and *b*_*MOX3*_. The second combination is extended from the first combination with another model based on *b*_*MD*_ (i.e. bouts detected from the output of the OC-Mahalanobis model *d*_*MD*_). The third combination includes models in the second combination and another model based on *b*_*GM*_ (i.e., bouts detected from the output of the OC-Gaussian model *s*_*GM*_). In summary, the three combinations are as follows:

• the models are based on *b*_*MOX1*_, *b*_*MOX2*_, and *b*_*MOX3*_;
• the models are based on *b*_*MOX1*_, *b*_*MOX2*_, *b*_*MOX3*_ and *b*_*MD*_;
• the models are based on *b*_*MOX1*_, *b*_*MOX2*_, *b*_*MOX3*_, *b*_*MD*_ and *b*_*GM*_.

**FIGURE 11:**
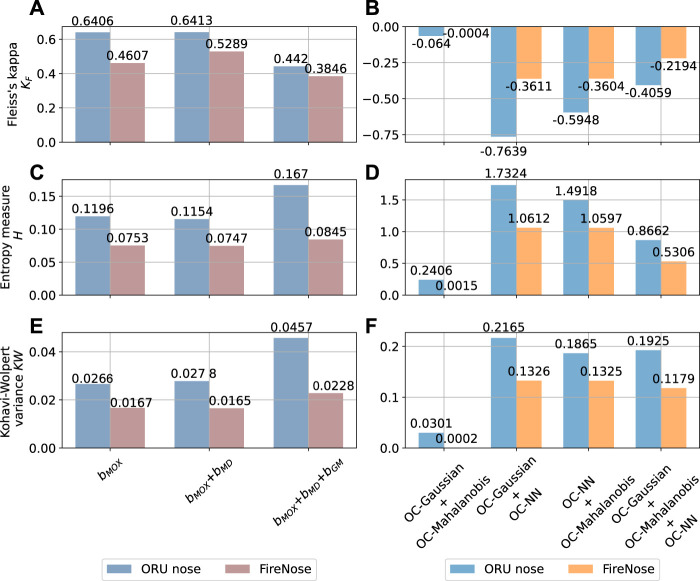
The change of the diversity before and after using model ensemble. The diversity is estimated with the Exp. 3-source trial using FireNose and ORU nose. Each bar corresponds to the diversity measure of a model combination. The composition of each combination can be found in the main text. **(A, C, E)** The values of Fleiss’ Kappa *K*_*F*_, entropy measure *H* and Kohavi-Wolpert variance *KW* of each base bout detection model combination for the FireNose and the ORU nose. **(B, D, F)** The values of Fleiss’ Kappa *K*_*F*_, entropy measure *H* and Kohavi-Wolpert variance *KW* of each one-class model combination for the FireNose and the ORU nose. The improvements of ensemble diversity are indicated by lower *K*_*F*_, higher *H* and higher *KW*.

The diversity measures of the three selected base model combinations are reported in Figures 11A,C,E for the Exp. 3-source trial using FireNose and ORU nose. With all three considered diversity measures, one can observe that the models in the third combination have smaller Fleiss’s Kappa *K*_*F*_, larger entropy measure *H* and larger Kohavi-Wolpert variance *KW* compared to the first and the second combinations is gained by using an ensemble of individual base models. The observations on the comparisons of *K*_*F*_, *H* and *KW* indicate a higher degree of diversity. Given this result, we demonstrate that stacking the signals of *d*_*MD*_ and *s*_*GM*_ and integrating them with the raw sensor responses into the ensemble model brings an improvement to the overall diversity.

### 4.2 Evaluation of the Individual One-Class Models

In the last subsection, we demonstrated that the Bout-Count Detection (BCD) model allows the detection of the presence of target analytes when there are significant changes in the raw sensor responses or in the output signals of the OC-Gaussian and the OC-Mahalanobis models. For the overall performance of the ensemble model, it is important to reliably distinguish between gas responses and baseline responses in case the sensor responses do not generate significant bouts. This situation might occur when sensor responses are at a steady-state. Although open sampling systems are likely to deal with fluctuating concentration levels, steady-state signals are still possible when gas sensors are exposed to concentration levels that are higher than the upper limits of the sensor sensing ranges. In this condition, sensor responses are only weakly dependent on gas concentrations and therefore exhibit a saturation behaviour in the form of steady-state signals (Satterthwaite et al., 2019). In practice, we also spotted that significant bouts did not appear even though sensor responses were not at a steady-state. For example, as shown in zoomed-in Figures 9, 14, between two detected overall bouts, there are segments of responses whose amplitude values are not significantly lower, which means these sensor responses probably correspond to gas exposures that should not be identified as clean air. In order to compensate for the BCD model, the OC-Gaussian, the OC-Mahalanobis models, trained on baseline responses, and the OC-NN model trained on the self-labelled gas responses, are introduced to process instantaneous measurements without relying on bout detection. This sub-section shows the diversity among these three predictive models from their outputs.

First of all, we show that the OC-NN model is an important contribution to improve ensemble diversity. We set up four base one-class model combinations and calculate Fleiss’ Kappa, entropy measure and Kohavi-Wolpert variance for each combination. The compositions of the four combinations are as follows:

• the OC-Gaussian model and the OC-Mahalanobis model are included;
• the OC-NN model and the OC-Mahalanobis model are included;
• the OC-NN model and the OC-Gaussian model are included;
• all the three one-class models are included.

We expected that the combinations that include the OC-NN model result in higher diversity measures. This hypothesis is validated in Figures 11B,D,F, which present the resulting diversity measures of the four model combinations with Exp. 3-source using the FireNose and the ORU nose. In each subplot, the corresponding diversity measure indicates that the OC-Gaussian model and the OC-Mahalanobis model lack diversity among each other. However, as long as the combination includes the OC-NN model, all the considered measures, i.e. Fleiss’ Kappa, entropy and Kohavi-Wolpert variance, indicate higher diversity. This result shows that the OC-NN model creates significant diversity for the one-class ensemble and matches our expectation on the OC-NN model since it is based on a different working principle and is learned from different training data. Although the three-model ensemble does not outperform the two-model ensemble (the OC-NN + the OC-Gaussian or OC-Mahalanobis), it is rather hard to exclude either the OC-GM model or OC-Mahalanobis model, because an exclusion requires prior knowledge on the target analytes. The OC-Mahalanobis model takes into the correlations between the sensor responses, whereas the OC-Gaussian model assumes gas sensors are independent with each other. However, given a priori unknown target analyte, we do not know to what extent the sensor responses are correlated. We are also aware of the risk that a poorly-learned OC-NN model might negatively affect the ensemble. This concern is addressed with a sensitivity analysis of the OC-NN model presented in the Supplementary Material.

**FIGURE 12:**
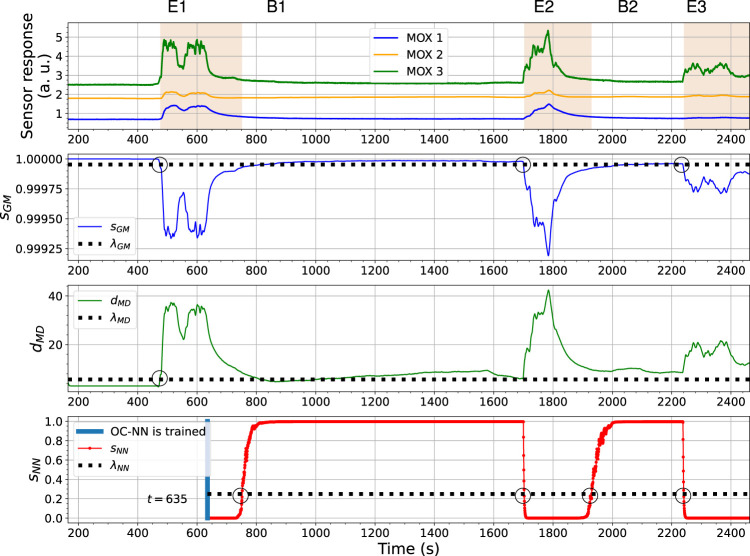
The output changes of the OC-Gaussian, the OC-Mahalanobis, and the OC-NN models using FireNose in the Exp. 3-source trial. The gas responses recognized by the ensemble model are in red shade in the sub-figure at the top. The meanings of E1, B1, E2, B2 and E3 are explained in the text.

Next, we demonstrate that the ensemble of the three individual models can perform gas detection without bout detection. Figure 12 presents an example prediction result on the same experimental trial as reported in Figure 7 (the Exp. 3-source trial using FireNose). The figure shows that how the individual models contribute to the final prediction. The overall output of the ensemble model for all three sensors is visualized in the subplot at the top in Figure 12, where sensor responses overlapping the periods shaded in red are recognized as significantly different from baseline responses. The respective predictions made from the *s*_*GM*_, *d*_*MD*_ and *s*_*NN*_ dynamics are shown below. As the e-nose started to be exposed to a target gas the first time, the output of the OC-Gaussian, *s*_*GM*_, began to decrease. At the same time, the output of the OC-Mahalanobis model, *d*_*MD*_, began to increase. According to the intersections between the thresholds *λ*_*GM*_ and *λ*_*MD*_ and the curves of *s*_*GM*_ and *s*_*MD*_, a first gas exposure is detected. The joint detection is marked as E1 in the figure. Provided gas responses are detected, the phase 2 model learning for the OC-NN model was then triggered. The OC-NN model is trained when a predefined number of gas responses are collected. In the shown case, learning of the OC-NN model lasted approximately 160 s, starting from around *t* = 473 s and ending at *t* = 633 s.

**FIGURE 13:**
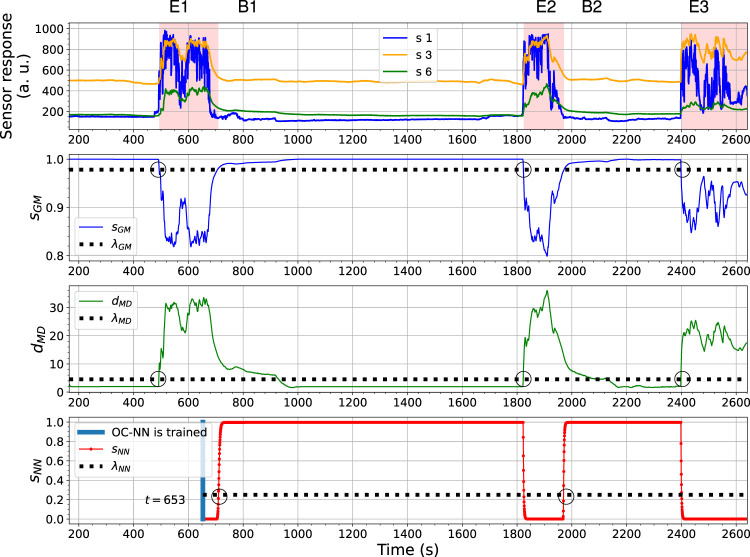
The outputs changes of the OC-Gaussian, the OC-Mahalanobis, and the OC-NN models using ORU nose in the Exp. 3-source trial. The gas responses recognized by the ensemble model are in red shade in the sub-figure at the top.

Once the OC-NN is finished learning (marked with a blue vertical line), its output *s*_*NN*_ score then began to indicate the likelihood of being clean air. *s*_*NN*_ first stayed at a low level because the gas exposure, from which it learned, was not finished yet. When the sensors did not exhibit significant responses, the OC-NN model successfully declared the sensor responses as clean air until the second gas exposure led to an increase of *s*_*NN*_. Note that the OC-NN model has “one vote veto”, and here the overall determination was made by the OC-NN (marked as B1) even though the OC-Gaussian and the OC-Mahalanobis models did not recognize clean air yet. When the sensors became exposed to a target analyte again (i.e. the second gas exposure), the *s*_*GM*_ and *d*_*MD*_ exceeded their corresponding thresholds, which determined the second gas detection (marked as E2). At around *t* = 1700 s, *s*_*NN*_ also fell below the decision threshold. At the end of the second gas exposure, the baseline responses were recognized by the OC-NN since *s*_*NN*_ went back to above *λ*_*NN*_ (marked as B2). Once again, the “one vote veto” by the OC-NN model is critical because the values of *s*_*GM*_ and *d*_*MD*_ still indicated gas responses at that time. At the beginning of the third gas exposure, all three models reacted as the sensor responses increase. *s*_*GM*_, *d*_*MD*_ and *s*_*NN*_ turned over their corresponding thresholds at a similar time. The detections made by the OC-Gaussian and the OC–Mahalanobis models are sufficient to declare the third gas exposure (marked as E3). Similar performances of the three models can be found in Figure 13, where the sensor responses of ORU nose are used.

In both results, the behaviour of the OC-NN model agrees with our previous investigation on the model diversity. The output *s*_*NN*_ is not highly correlated with *s*_*GM*_ and *d*_*MD*_, since the OC-NN model is learned on gas responses instead of baseline responses, whereas the corresponding models of *s*_*GM*_ and *d*_*MD*_ are trained with baseline responses. The diversity among models is supposed to be beneficial for an ensemble model. An observed benefit is that the OC-NN model recognizes clean air faster than the OC-Gaussian and the OC-Mahalanobis models. In other words, in the presented trial, the short-term sensor drift between gas exposures, likely caused by the long recovery time of the sensors (e.g., shown in Figure 2C), is addressed by the ensemble modelling, mainly owing to the comparatively robust prediction from the OC-NN. In this trial, compared to the performance of the OC-Gaussian and OC-Mahalanobis models, the OC-NN model seems to be less sensitive to the precise value of the selected threshold.

**FIGURE 14:**
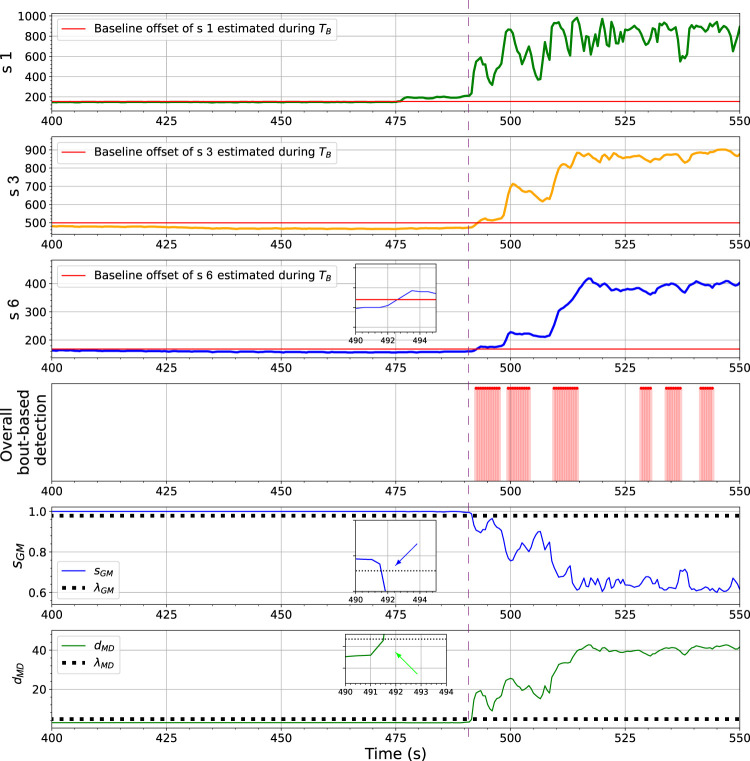
The changes of ORU nose sensor responses, *s*_*GM*_, *d*_*MD*_ and the BCD model output towards the first gas exposure in the Exp. 3-source trial. The vertical purple dashed line locates the time point where *s*_*GM*_ and *d*_*MD*_ presented significant indications of gas detection (as pointed by corresponding arrows) when sensor responses of *s* 2 and *s* 3 are still below their baseline offset values. One can clearly observe that at *t* = 492 s, *s*_*GM*_ and *d*_*MD*_ are their thresholds while the values of *s* 2 and *s* 3 are below their corresponding baseline offsets.

The ensemble learning-based gas detection system aims to recognize gas responses suitable for the consequent discriminative process rather than aiming for the fastest possible detection. One can observe that before the beginning of the first gas exposure in Figure 14, a short range of the rising phase in the sensor response of *s* 1, between *t* = 475 s to *t* = 490 s, has not been caught by any proposed one-class models. During this period, the sensors were likely in contact with gas patches released from the source. However, since the response amplitudes of *s* 3 and *s* 6 are lower than the corresponding baseline offsets in this period, it makes sense to discard these onset periods. The fingerprints of these instantaneous measurements in the feature space are not informative enough to be distinguished by models based on one-class Gaussian (OC-Gaussian), Mahalanobis distance (OC-Mahalanobis), or proximity between nearest neighbours (OC-NN). Accordingly, such measurements are prone to have poor class separability for gas discrimination.

We observed similar results for the Exp. 2-source trial, which are reported in the Supplementary Material.

## 5 Conclusion and Future Work

This paper proposes an ensemble learning-based gas detection approach that reduces the need for prior knowledge about the target analytes. The proposed approach consists of two model learning phases. The first phase allows a one-class Gaussian (OC-Gaussian) model and a one-class Mahalanobis distance-based (OC-Mahalanobis) model to be trained with baseline responses. In a parallel manner, a bout-count detection (BCD) model extracts bouts from raw sensor responses as well as the outputs of the OC-Gaussian and the OC-Mahalanobis models, and learns a one-class model based on bout detection of these five signals (two model outputs and three sensor responses). Gas responses recognized by the OC-Gaussian, the OC-Mahalanobis, and the BCD models trigger phase 2 model learning. A one-class nearest neighbour (OC-NN) model is trained with self-labelled gas responses in phase 2 learning. We presented a diversity evaluation on the used models and demonstrated how ELBA performs gas detection with the data sets produced by real-world experiments.

The proposed approach is not optimized to overcome the long recovery time (recognize clean air measurements before the responses are fully recovered), which is a limitation compared to sensor modelling approaches that aim to address this issue, e.g. (Monroy et al., 2012), and (Eu and Yap, 2014).

Regarding the future work, the ELBA algorithm could be improved in two aspects as follows:

• We will have a thorough investigation to validate the BCD model. The original idea of including the bout-detection models is to consider the situations where the one-class models might learn their corresponding thresholds too high. When these situations occur, the one-class models might be unable to recognize gas responses from *s*_*GM*_, *d*_*MD*_, *s*_*NN*_ values. The BCD model is supposed to compensate for such situations with the assumption that gas responses come with transient signals in the form of bouts. However, the presented experimental trials did not replicate the above situations, so the potential value of the BCD model is not demonstrated well. A future evaluation will base on data sets that are challenging for the other one-class models to learn appropriate thresholds and, therefore, verify the contribution of the BCD model to the final prediction.
• Since the ELBA approach includes several free parameters that need to be determined empirically, sensitivity to the parameter selection should be fully tested. The detection performance will be further evaluated the measures such as true alarm rate, false alarm rate, and delay of detection. The experiments will consider more types of gases, and the experimental parameters such as sensor heating time, the time and distance of the interaction between the gas source and the e-nose strictly controlled. In particular, a photoionization detector should be applied to provide calibrated concentration values as ground-truth. In the current presented work, we can only assess how the ELBA behaves on the sensor responses, which does not allow accurate evaluation in terms of true/false alarm rate. With calibrated ground-truth, we can address the potential performance degrade when the later exposure(s) is different from the first exposure in gas identity. As proof of the concept, we demonstrate that the OC-NN models can learn from the first gas exposure and handle exposures of other gases. In the future, gas classes will be considered for detection. For example, a gas discrimination algorithm can be coupled with the ELBA approach, which allows to learn a dedicated threshold for each appeared gas class. In this way, the gas detection procedure can further declare the presence of new, unseen gas classes, which is not addressed in the current work.
• For applications requiring fast detection, we can modify the voting scheme to reacts faster to changes in instantaneous responses and adjust the BCD model to be more sensitive to small bouts. The improvement in this direction will allow the ELBA algorithm to be used as an early exposure detector.
• The proposed approach could be extended to include a dedicated model for drift elimination. For example, the methods introduced in (Martinelli et al., 2013) and (Magna et al., 2018) can be coupled to the modes for baseline responses in the ensemble. The idea of adding a model for drift elimination is to particularly learn the pattern of the drift responses and, therefore, to allow adaptive drift compensation.
• The ensemble structure of the ELAB approach allows an adaption to integrate supervised learning models in order to better tackle problems where not all gases are a priori unknown. In practice, depending on the availability of the prior knowledge on the target analytes, the restriction to using one-class learning for gas detection can be relaxed. This type of problem setup opens the possibility to consider hybrid supervised and unsupervised learning for gas detection. A hybrid system seeks to balance the need for accurate detection of known gases and the adaptivity enabled by retrieving new information from acquired measurements.

## Data Availability

The data analyzed in this study is subject to the following licenses/restrictions: The dataset was generated via a joint work with collaborators from the University of Warwick. The request of using the ad hoc dataset will be approved with the permission of the authors and collaborators of the University of Warwick. Requests to access these datasets should be directed to han.fan@oru.se.
