# Supplementary material for: Ensemble Learning-Based Approach for Gas Detection Using an Electronic Nose in Robotic Applications
Source: Front Chem. 2022 Apr 28;10:863838. doi: 10.3389/fchem.2022.863838 (PMC9096169; doi:10.3389/fchem.2022.863838)
Supplement: Supplementary file 1 [file DataSheet1.PDF]

Supplementary Material

1 SENSITIVITY ANALYSIS OF THE OC-NN MODEL

The One Class Nearest Neighbour (OC-NN) model is learned from baseline responses and self-labelled gas responses, which are associated with the predictive behaviour of the OC-NN model. As is known, the predictive performance of a machine learning model often improves with the extensiveness of the training data. In this case, intuitively, if the self-labelled gas responses used for training only contain high-concentration measurements, the learned OC-NN model has a risk to recognize measurements with lower concentration levels as clean air. This section provides a sensitivity analysis to quantify the output changes of the OC-NN model learned under varying conditions. This sensitive analysis focuses on evaluating how the concentration levels of the training data affect the prediction of the OC-NN model.

The first step of the sensitivity analysis is to define a test harness to evaluate model behaviour and then evaluate the OC-NN model on the same test data with different learning data. Given a set of gas responses $\mathbf{X}_L$, a training data set $\mathbf{X}_q$ is configured by extracting measurements whose concentration indicator I_C is above the q-th quantile of $\mathbf{X}_L$. The concentration indicator I_C is an approximation of the concentration level using sensor responses. In this work, it is defined as the mean value of the three instantaneous sensor responses of the ORU nose or the FireNose. In total 5 training data sets are prepared: $\mathbf{X}_0$, $\mathbf{X}_{0.1}$, $\mathbf{X}_{0.2}$, $\mathbf{X}_{0.4}$ and $\mathbf{X}_{0.8}$.

Second, for each training set $\mathbf{X}_q$, an OC-NN model is learned and then is tested with a common test set $\mathbf{X}_T$. The OC-NN model will calculate a s_{NN} value for each measurement in $\mathbf{X}_T$.

The estimated mean and standard deviation of the s_{NN} values corresponding to different OC-NN models are evaluated in Fig. S1 and Fig. S2 for the 2-source trials and the 3-source trials, respectively. In both trials, the set $\mathbf{X}_L$ contains gas responses in the first gas exposure and the testing data $\mathbf{X}_T$ are gas responses corresponding to the later gas exposures.

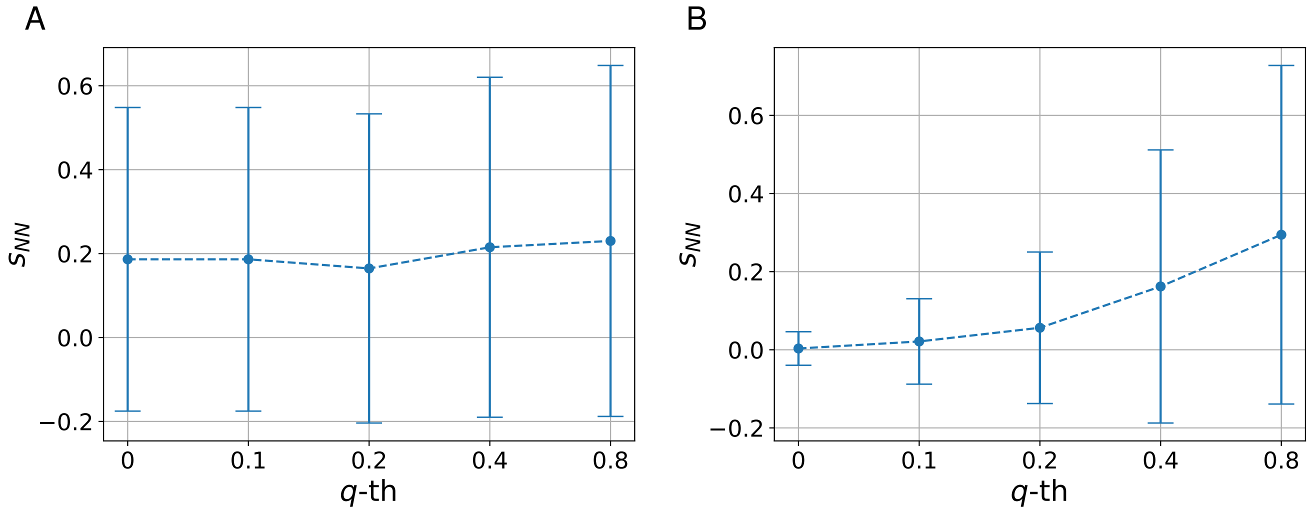

Figure S1. The estimated mean and standard deviation of the s_{NN} obtained with OC-NN models learned from gas responses of different concentration levels. The training data sets X_q, and the testing data X_T are from the 2-source experimental trial using **(A)** the ORU nose and **(B)** the FireNose.

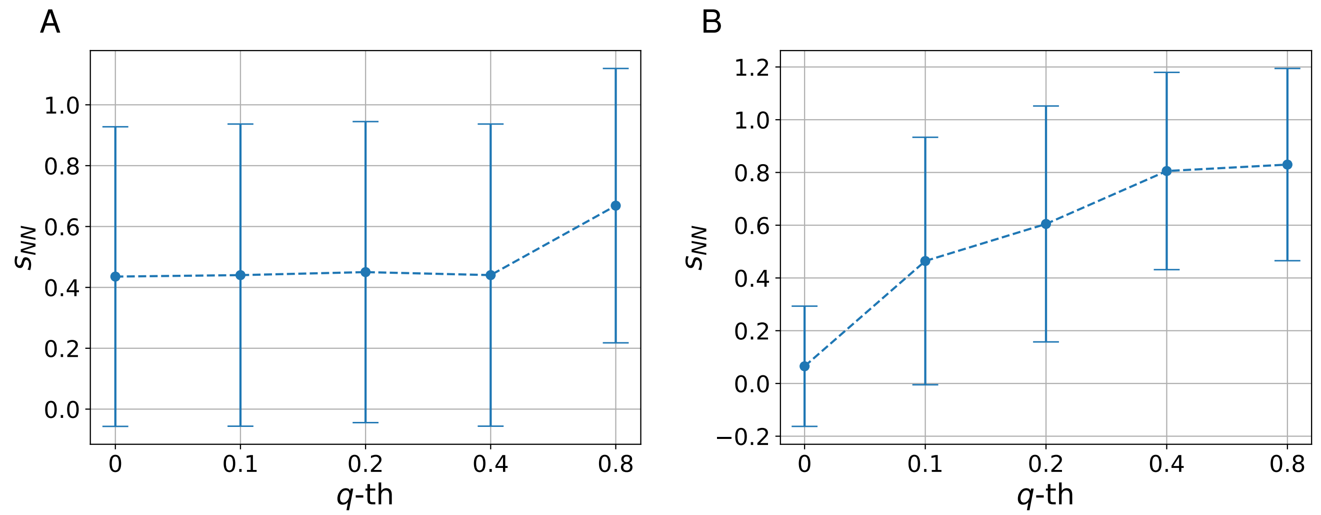

Figure S2. The estimated mean and standard deviation of the s_{NN} obtained with OC-NN models learned from gas responses of different concentration levels. The training data sets X_q, and the testing data X_T are from the 3-source experimental trial using **(A)** the ORU nose and **(B)** the FireNose.

From the results we can confirm that the OC-NN models learned with less low-concentration gas responses is prone to have missed detection on gas responses since the predictive model assign higher s_{NN} values to the measurements in $\mathbf{X}_T$. For the ORU nose, this issue seems to be less critical since the corresponding OC-NN model was sensitive to the configuration of the learning data in both trials (the outputted s_{NN} are stable among the models learned with X_0, $X_{0.1}$ $X_{0.2}$, and $X_{0.4}$).

2 THE PERFORMANCE OF THE ELBA APPROACH ON THE 2-SOURCE EXPERIMENTAL TRIAL

The performance of the ELBA approach on the 2-source experimental trial is shown in Fig. S3 and Fig. S4. In general, the performance of the OC-Gaussian and the OC-Mahalanobis models in this trial are quite similar with those in the 3-source trial. The outputs of the OC-NN model with both e-noses exhibited more fluctuations around $t = 950$ s and $t = 1400$ s. The OC-NN model with the ORU nose did not immediately recognize the baseline responses after $t = 1350$ s (at which time the robot has been already more than 4 meters away from the second gas source). Nevertheless, the OC-Gaussian and the OC-Mahalanobis models agreed to declare the presence of clean air.

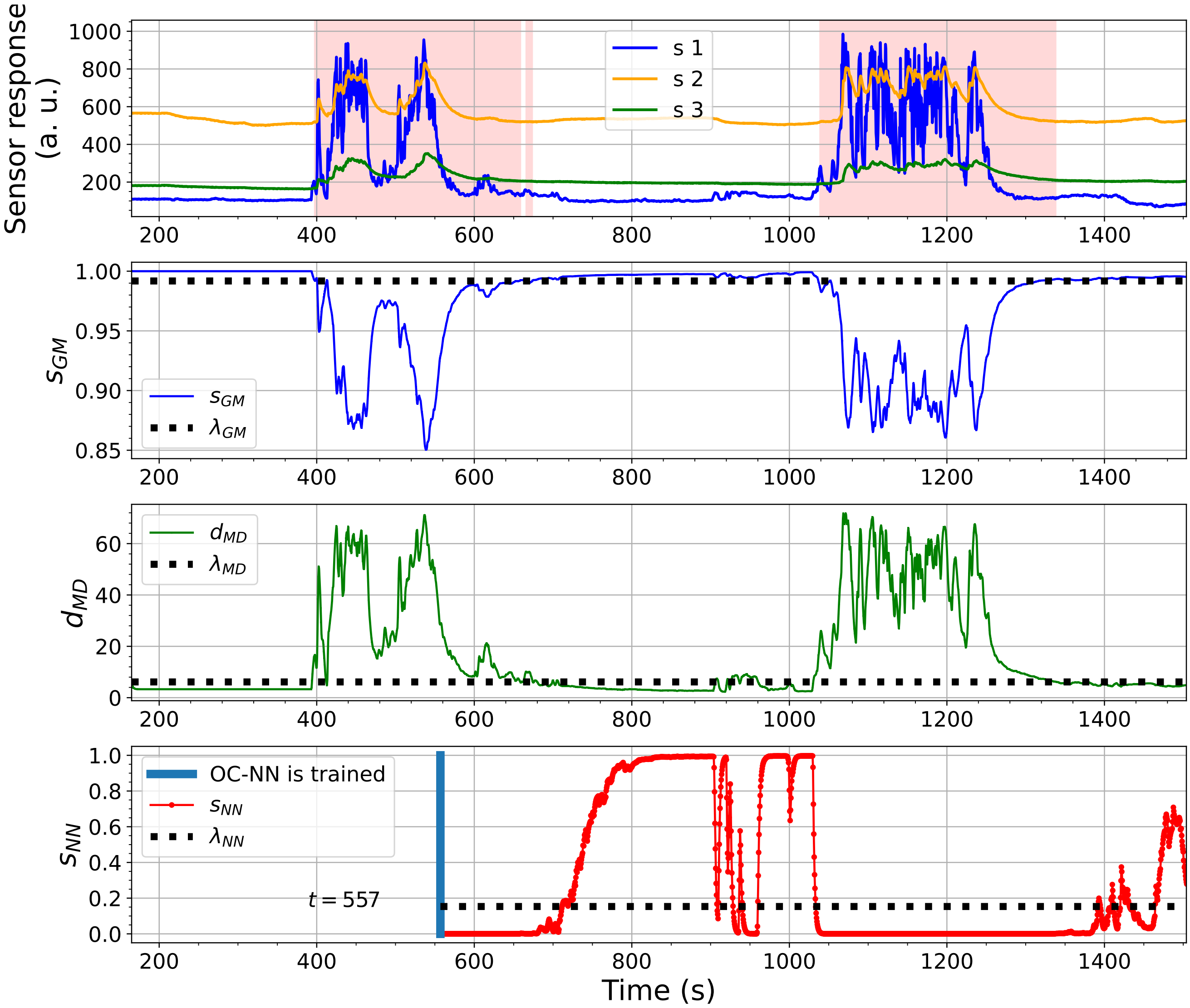

Figure S3. The outputs changes of the OC-Gaussian, the OC-Mahalanobis, and the OC-NN models using ORU nose in the Exp. 2-source trial. The gas responses recognized by the ensemble model are in red shade in the sub-figure at the top.

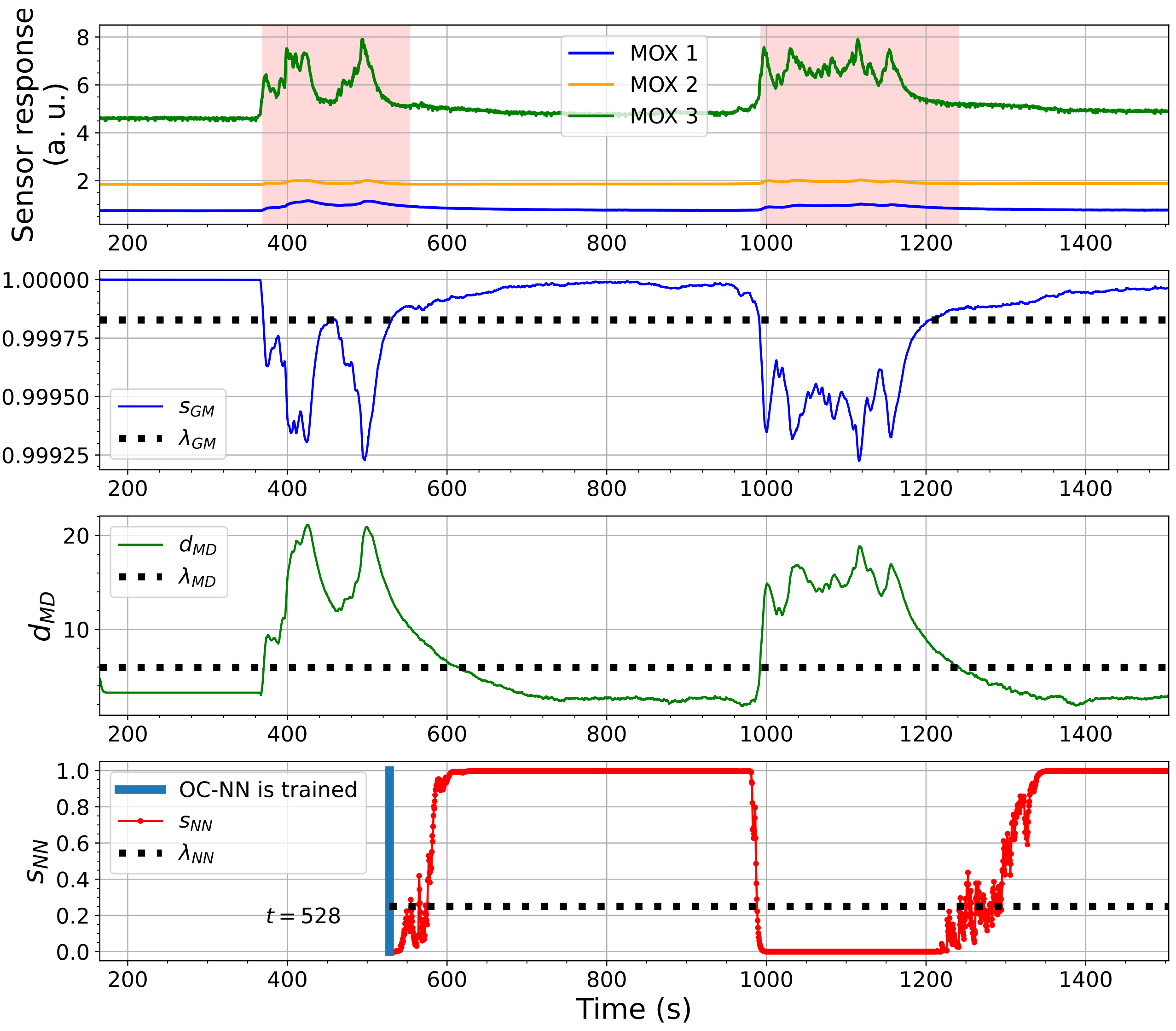

Figure S4. The outputs changes of the OC-Gaussian, the OC-Mahalanobis, and the OC-NN models using ORU nose in the Exp. 2-source trial. The gas responses recognized by the ensemble model are in red shade in the sub-figure at the top.
